# Blue light induces major changes in the gene expression profile of the cyanobacterium *Synechocystis* sp. PCC 6803

**DOI:** 10.1111/ppl.13086

**Published:** 2020-03-14

**Authors:** Veerle M. Luimstra, J. Merijn Schuurmans, Klaas J. Hellingwerf, Hans C. P. Matthijs, Jef Huisman

**Affiliations:** ^1^ Department of Freshwater and Marine Ecology, Institute for Biodiversity and Ecosystem Dynamics University of Amsterdam Amsterdam The Netherlands; ^2^ Wetsus – Center of Excellence for Sustainable Water Technology Leeuwarden The Netherlands; ^3^ Swammerdam Institute for Life Sciences University of Amsterdam Amsterdam The Netherlands

## Abstract

Although cyanobacteria absorb blue light, they use it less efficiently for photosynthesis than other colors absorbed by their photosynthetic pigments. A plausible explanation for this enigmatic phenomenon is that blue light is not absorbed by phycobilisomes and, hence, causes an excitation shortage at photosystem II (PSII). This hypothesis is supported by recent physiological studies, but a comprehensive understanding of the underlying changes in gene expression is still lacking. In this study, we investigate how a switch from artificial white light to blue, orange or red light affects the transcriptome of the cyanobacterium *Synechocystis* sp. PCC 6803. In total, 145 genes were significantly regulated in response to blue light, whereas only a few genes responded to orange and red light. In particular, genes encoding the D1 and D2 proteins of PSII, the PSII chlorophyll‐binding protein CP47 and genes involved in PSII repair were upregulated in blue light, whereas none of the photosystem I (PSI) genes responded to blue light. These changes were accompanied by a decreasing PSI:PSII ratio. Furthermore, many genes involved in gene transcription and translation and several ATP synthase genes were transiently downregulated, concurrent with a temporarily decreased growth rate in blue light. After 6–7 days, when cell densities had strongly declined, the growth rate recovered and the expression of these growth‐related genes returned to initial levels. Hence, blue light induces major changes in the transcriptome of cyanobacteria, in an attempt to increase the photosynthetic activity of PSII and cope with the adverse growth conditions imposed by blue light.

**Abbreviations**
ATP: adenosine triphosphate
Chl *a*: chlorophyll *a*
Hik: histidine kinase
LED: light‐emitting diode
NADPH: reduced nicotinamide adenine dinucleotide phosphate
PBS: phycobilisomes
PC: phycocyanin
PSI: photosystem I
PSII: photosystem II

## Introduction

Cyanobacteria play a key role in aquatic ecosystems and are widely hailed as the evolutionary ancestors of chloroplasts and thus are often used as model organisms to study oxygenic photosynthesis. Similar to eukaryotic photosynthetic organisms, cyanobacteria use the ubiquitous pigment chlorophyll *a* (Chl *a*) in photosystem I (PSI) and photosystem II (PSII). Chl *a* absorbs in the blue part (peak wavelength at ~440 nm) and red part (680 nm) of the light spectrum (Engelmann 1882, Kirk 2011). In addition, cyanobacteria deploy a diversity of phycobili‐pigments in large light‐harvesting antennae, known as phycobilisomes (PBS). These phycobili‐pigments include phycourobilin, phycoerythrobilin and phycocyanobilin, which have absorption maxima in cyan (495 nm), green (545 nm) and orange light (625 nm), respectively (Tandeau de Marsac 2003, Six et al. 2007).

Hence, cyanobacteria absorb blue light ≤450 nm. Yet, contrary to green algae and plants, PBS‐containing cyanobacteria have much lower rates of photosynthesis and growth in blue light than in the other light colors absorbed by their photosynthetic pigments (Lemasson et al. 1973, Pulich and van Baalen 1974, Wyman and Fay 1986, Jørgensen et al. 1987, Wilde et al. 1997, Tyystjärvi et al. 2002, Wang et al. 2007, Singh et al. 2009, Chen et al. 2010, Choi et al. 2013, Bland and Angenent 2016, Luimstra et al. 2018). As a consequence, PBS‐containing cyanobacteria can be strong competitors in cyan, green or orange light absorbed by their PBS, but they are very poor competitors in blue light ≤450 nm in comparison to photosynthetic organisms with chlorophyll‐based light‐harvesting complexes (Luimstra et al. 2020). The cyanobacterium *Prochlorococcus* is an interesting exception. *Prochlorococcus* lacks PBS but instead uses divinyl‐chlorophyll‐based light‐harvesting complexes quite similar to green algae and terrestrial plants, and it performs very well in blue light as it is the most abundant cyanobacterium in the blue waters of the open ocean (Chisholm et al. 1988, Flombaum et al. 2013).

Why PBS‐containing cyanobacteria have a low photosynthetic efficiency in blue light is not yet fully resolved, but most likely it can be attributed to an imbalance of the light energy captured by PSI and PSII. In contrast to green algae and plants, PBS‐containing cyanobacteria invest much more of their Chl *a* in PSI than in PSII (e.g. Myers et al. 1980, Fujita 1997, Luimstra et al. 2018). As a consequence, PSI will absorb more blue light than PSII. Conversely, the PBS of cyanobacteria transfer most of their absorbed light energy to PSII (Joshua et al. 2005, Mullineaux 2014, Kirilovsky 2015). However, the phycobili‐pigments of PBS do not absorb blue light ≤450 nm (Grossman et al. 1993, Tandeau de Marsac 2003, Six et al. 2007). Hence, especially under light‐limited conditions, blue light provides sufficient excitation energy at PSI but insufficient excitation energy at PSII to sustain high rates of linear electron flow (Fujita 1997, Solhaug et al. 2014, Kirilovsky 2015, Luimstra et al. 2018, 2019).

Several recent observations support the above‐mentioned explanation for the low photosynthetic efficiency of PBS‐containing cyanobacteria in blue light. At low light intensities, oxygen production rates of *Synechocystis* sp. PCC 6803 were much lower in blue than in orange and red light, whereas at high light intensities the maximum oxygen production rates were similar in all three light colors (Luimstra et al. 2018). Furthermore, several studies have shown that the PSI:PSII ratio of PBS‐containing cyanobacteria strongly decreases in blue light (Wilde et al. 1997, El Bissati and Kirilovsky 2001, Singh et al. 2009, Luimstra et al. 2018, 2019). Finally, a PBS‐deficient mutant of *Synechocystis* sp. PCC 6803 showed a similar low photosynthetic efficiency in both blue and orange‐red light, comparable to the PBS‐containing wild‐type in blue light (Luimstra et al. 2019). These observations are all consistent with the hypothesis that the low absorption of blue light by the light‐harvesting PBS, and hence the limited transfer of excitation energy to PSII, is the rate‐limiting factor for photosynthesis in blue light.

In view of these strong photo‐physiological responses, we hypothesize that PBS‐containing cyanobacteria will display major changes in their gene expression profile when exposed to blue light. In particular, if blue light causes an imbalance between PSI and PSII, one may expect upregulation of PSII genes and downregulation of PSI genes. Furthermore, preferential excitation of PSI may lead to enhanced ATP production through cyclic electron flow without reduced nicotinamide adenine dinucleotide phosphate production (Allen 2003, Munekage et al. 2004, Yeremenko et al. 2005), which is likely to induce major shifts in the expression of many genes involved in the metabolic pathways that are dependent on the photosynthetic activity of the cells. Yet, although several studies have investigated how changing light conditions affect cyanobacterial gene expression (e.g., Hihara et al. 2001, Gill et al. 2002, Huang et al. 2002, Billis et al. 2014, Xiong et al. 2015), the transcriptome response of cyanobacteria to different colors of light has received much less attention (but see Singh et al. 2009 and Hübschmann et al. 2005).

In this study, we therefore investigate how blue, orange and red light affect the gene expression profile of *Synechocystis* sp. PCC 6803. This PBS‐containing model cyanobacterium was grown in light‐limited chemostats and acclimated to ‘artificial white’ light (or more precisely, polychromatic light with an equal mix of blue, orange and red photons). When the chemostats reached steady‐state, the incident light was switched to monochromatic blue, orange or red light. The transcriptomes were analyzed just before and at several time points after this switch in light color, until a new steady‐state was reached. Hence, our transcriptome analysis allowed investigation of changes in gene expression associated with transient cellular processes as well as with long‐term acclimation to the three different light colors. In line with our hypothesis, the switch to blue light had a much stronger effect on the transcriptome than the switch to red and orange light, consistent with a cellular effort to restore the excitation balance between PSI and PSII. This striking difference in regulatory responses to different light colors adds further insight into the photosynthetic traits of cyanobacteria, which is of relevance for both biotechnological and ecological applications.

## Materials and methods

### Strains and culture conditions

All experiments were performed using the cyanobacterium *Synechocystis* sp. PCC 6803, which was kindly provided by D. Bhaya (University of Stanford, USA). *Synechocystis* sp. PCC 6803 was cultured in flat‐walled 1.8‐l chemostats as described by Huisman et al. (2002). These chemostats allow full control of light conditions, CO_2_ and nutrient inflow, and temperature. Light was provided by narrow‐band light‐emitting diode panels with a full width at half maximum of ~20 nm (Philips Lighting B.V., The Netherlands) at a total incident light intensity (*I*_in_) of 60 μmol photons m^−2^ s^−1^. Light passing through the chemostats (*I*_out_) was measured using a LI‐250 light meter (LI‐COR Biosciences, USA). The cultures were provided with a nutrient‐rich mineral medium (BG‐11 medium; Merck, Germany) supplemented with 5 mM Na_2_CO_3_ and were maintained at a dilution rate of *D* = 0.03 h^−1^. Temperature was kept constant at 30°C. CO_2_‐enriched air (2% v/v) flowing at a rate of 30 l h^−1^ was used to provide CO_2_ and to keep the culture mixed. The CO_2_ concentration of the gas influx was regularly monitored using an Environmental Gas Monitor for CO_2_ (EGM‐4; PP Systems, USA).

### Chemostat experiments

Nine chemostats were illuminated with ‘artificial white’ light, consisting of an even mixture of LEDs emitting blue (450 nm), orange (625 nm) and red light (660 nm). Each of these light colors was provided at 20 μmol photons m^−2^ s^−1^, reaching a total *I*_in_ of 60 μmol photons m^−2^ s^−1^ until steady‐state was reached after 5–6 volume changes. After steady‐state was reached, the light color was switched to 60 μmol photons m^−2^ s^−1^ of either monochromatic blue, orange or red light. Each light color was applied to three chemostats, providing three independent biological replicates per color for cell counts, absorbance, fluorescence and transcriptome analyses.

Cell counts in the chemostats that were switched to blue light declined strongly, and became much lower than those in orange and red light. The lower cell numbers imply that cells in blue light were exposed to a higher average photon dosage per cell than cells in red and orange light, which may affect their physiology and gene expression. To distinguish between effects of light color and light intensity, we therefore also ran a series of chemostats (n = 3) in red light with a higher dilution rate (0.06 h^−1^), to obtain a similar dilute cell culture as in the chemostats exposed to blue light. These will be referred to as the ‘dilute chemostats in red light’.

### Sampling and measurements

Sampling occurred directly before (t = 0 h) and at 1, 2, 4, 8, 24, 48, 72, 96, 168, 192 and 216 h after the light switch. Cell counts and biovolume were measured on the day of sampling using a CASY 1 TTC cell counter with a 60 μm capillary (Schärfe Systems GmbH, Germany). Samples were measured in triplicate and diluted to ~5 × 10^4^ cells ml^−1^ in Casyton solution.

Ultraviolet/visible absorbance spectra were measured directly after sampling using an updated Aminco DW2000 photospectrometer (OLIS, USA). Absorbance was measured from 400 to 750 nm and normalized to maximum absorbance at 440 nm, after baseline correction for minimum absorbance at 750 nm.

To measure low‐temperature (77 K) fluorescence spectra, 1.5 ml of each sample was transferred to 3 ml‐cuvettes prefilled with 1.5 ml 60% glycerol and mixed by pipetting up and down three times. Cuvettes were flash‐frozen in liquid nitrogen and stored at −80°C. Fluorescence at 77 K was measured within a week from sampling with an OLIS DM45 spectrofluorometer (OLIS, USA) equipped with a Dewar cell. Fluorescence emission was measured from 630 to 750 nm with excitation at 440 nm (mainly Chl *a*) and 590 nm (mainly PBS). The final glycerol concentration in the cuvettes did not exceed 30% to minimize dissociation of PBS from the thylakoid membranes, and cell concentrations were below 4 × 10^7^ cells ml^−1^ to minimize re‐absorption of the fluorescence signal (Mao et al. 2003).

To guarantee that the fluorescence spectra reflected the photophysiology of the cells at the time of sampling, samples for the 77 K fluorescence analyses were processed and frozen within 20 seconds from the moment of sampling. Consequently, the photosynthetic proteins were fixed in the position they were in at the moment of sampling. As absorbed excitation energy can still be transferred between associated pigments, the heights of the fluorescence emission peaks can be used to estimate the relative contents and interactions of PSI and PSII and their associated PBS (e.g. Murakami 1997, Schuurmans et al. 2017). Our transcriptome results indicate that the expression of PSI genes remained rather stable while expression of PSII genes was regulated. Therefore, fluorescence emission by PBS (at 650 nm) and PSII (at 695 nm) was quantified relative to the fluorescence emission by PSI (at 725 nm).

### Microarray analysis

To investigate light‐color dependent differences in gene expression, total RNA was sampled from each chemostat (n = 3 per light color). Because large changes in physiology were observed within the first hours after the switch to blue light, RNA samples were collected from the blue‐light chemostats at t = 0, 1, 4, 24, 48 and 192 h and from the red‐ and orange‐light chemostats at t = 0 h (just before the switch), t = 4 h (during acclimation) and t = 192 h (in steady‐state). Additionally, an RNA sample was collected from the dilute chemostat in red light at t = 192 h. The 50 ml samples were centrifuged at 3000*g* at 4°C for 10 min. Cell pellets were immediately frozen in liquid nitrogen after resuspension in 1 ml TRIzol (Life Technologies, USA) and stored at −80°C. RNA extraction was performed using the Direct‐Zol™ RNA MiniPrep kit (Zymo Research, USA), including the in‐column DNAse I treatment. All samples had A_260_/A_280_ and A_260_/A_230_ values above 1.8 as quantified using a Nanodrop ND‐1000 spectrophotometer (Thermo Scientific, USA). RNA integrity was verified using an Agilent 2100 Bioanalyzer (Agilent Technologies, The Netherlands).

Custom‐made 45–65‐mer oligonucleotide microarrays were designed as described by Aguirre von Wobeser et al. (2011; see also Eisenhut et al. 2007, Krasikov et al. 2012) and printed on a 1 × 3 inch glass slide in a standard 2 × 11 K format by Agilent. All 3264 genes of *Synechocystis* PCC 6803 described in CyanoBase (http://genome.microbedb.jp/CyanoBase) were represented in the array design. We used 1–4 different probes per gene. Cy3‐ and Cy5‐labeled cDNA was generated from the total RNA with the Superscript III Reverse Transcriptase kit (Invitrogen Life Science, The Netherlands) and hybridized to the arrays as described by Eisenhut et al. (2007) using a loop design (Table S1).

The microarray data were analyzed with the *limma* package (version 3.24.14; www.bioconductor.org; Ritchie et al. 2015) using R version 3.2.1. (www.R-project.org), following the same approach as Schuurmans et al. (2018). First, background correction was applied to the raw array intensity data according to the *normexp* + *offset* = 20 method (Ritchie et al. 2007). Subsequently, within‐array normalization was applied using the *global loess* method and between‐array normalization was applied using the *A‐quantile* method (Smyth and Speed 2003). The *avedups* function was used to average replicate spot intensities (Smyth et al. 2005). All gene expression data were compared to expression in the chemostat cultures acclimated to artificial white light at t = 0 h using the linear modeling approach function *lmfit* (Smyth 2004). The empirical Bayes method incorporated in the *eBayes* function was used to evaluate the significance of changes in gene expression. For this purpose, we averaged the log_2_ ratios of different probes for the same gene and we used Fisher's method in the *metap* package (version 0.6) of R to combine the adjusted *P*‐values of these probes. Genes expressed with a log_2_ fold change of <−1.4 or >1.4 and adjusted *P*‐values <0.05 were considered differentially expressed. Heatmaps of the differentially expressed genes were made with the *Heatmap.2* function from the *gplot* package (version 2.17.0), and the *hclust* function was applied for hierarchical clustering of the genes based on Euclidean distances.

## Results

### Growth of *Synechocystis* in blue, orange and red light

**Figure 1:**
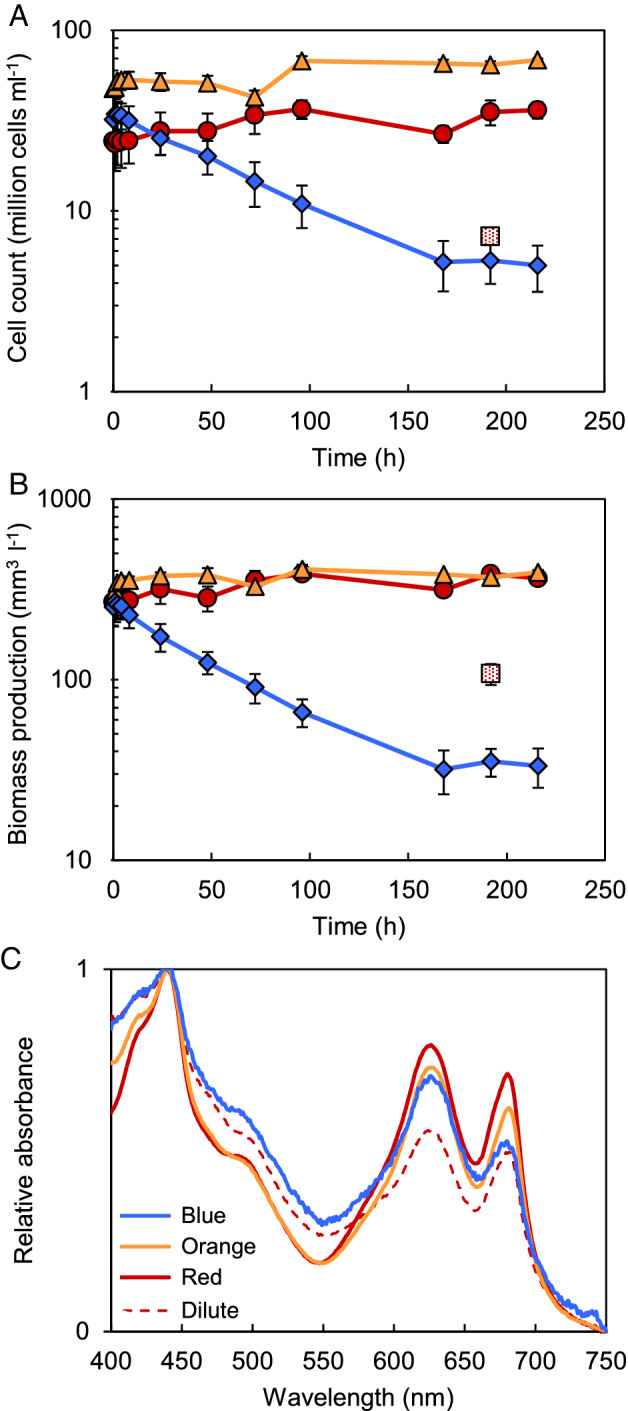
Cell and biomass production in light‐limited chemostats of *Synechocystis* sp. PCC 6803, after a switch from artificial white light to monochromatic blue, orange and red light. (A) Cell counts and (B) biomass obtained during growth in blue (diamonds), orange (triangles) and red light (circles). The red‐dotted square indicates the steady‐state cell count and biomass of the dilute chemostat in red light. Data are averages of three biological replicates ± SD (n = 3). (C) Light absorption spectra of the cells after acclimation to blue (blue line), orange (orange line) and red light (red line). The dashed red line indicates absorption by the dilute chemostat in red light. Samples were taken after 192 h, when chemostats were in steady‐state. The spectra were normalized to maximum absorbance at 440 nm, after baseline correction for minimum absorbance at 750 nm. Spectra are averages of three biological replicates (n = 3); SD averaged over the range from 400 to 750 nm was 0.036 in blue light, 0.007 in orange light, 0.033 in red light, and 0.013 in the red dilute chemostat.

The experiments were started by switching the wavelength of the light provided to steady‐state chemostats with *Synechocystis* sp. PCC 6803 from artificial white light to either blue, orange or red light. In orange and red light, cell counts and biomass were not strongly affected by this switch in light color (Fig. 1A,B). In blue light, however, cell counts and biomass decreased by ~85% and a new steady‐state was reached after ~168 h, with 5.2 ± 1.5 million cells ml^−1^ (Fig. 1A) and a total biomass (expressed as biovolume) of 33.5 ± 8.0 mm^3^ l^−1^ (Fig. 1B).

To distinguish between effects of light color and light intensity, we also ran a series of chemostats in red light with a higher dilution rate (0.06 h^−1^). These dilute chemostats in red light obtained a steady‐state cell density of 5.1 ± 0.6 million cells ml^−1^ (red‐dotted square in Fig. 1B), which is comparable to that of the blue‐light chemostats.

### Blue light induces changes in pigmentation and photosystem ratios

Absorption spectra measured at steady state of the chemostats revealed subtle differences in pigmentation of the cells (Fig. 1C). The phycocyanin (PC):Chl *a* ratio (relative absorption by PC at 625 nm vs Chl *a* at 680 nm) was highest in cells grown in blue light, indicating that *Synechocystis* sp. PCC 6803 produced more PC relative to Chl *a* in blue light than in orange and red light. Furthermore, the ratio of absorption at 440 nm (absorption by both Chl *a* and carotenoids) vs 680 nm (absorption by Chl *a*) indicates that it produced more carotenoids relative to Chl *a* in blue light and in the dilute chemostat in red light than in the other chemostats (Fig. 1C).

**Figure 2:**
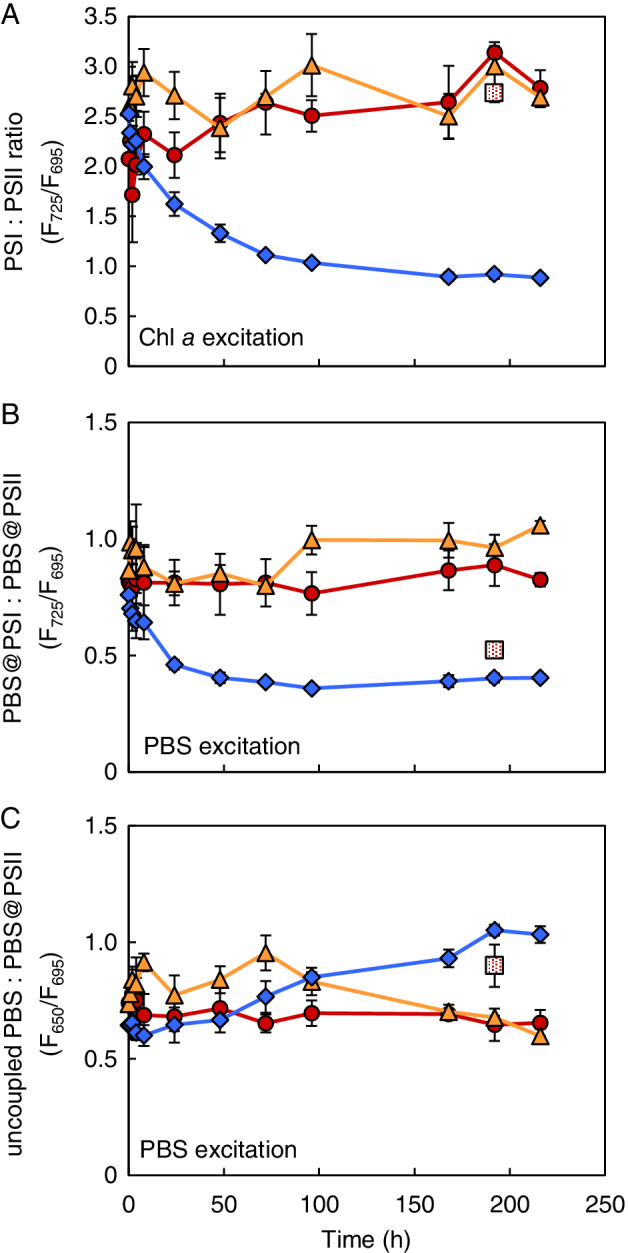
Ratios of PSI, PSII and PBS fluorescence emission peaks measured at 77 K of *Synechocystis* sp. PCC 6803, after a switch from artificial white light to monochromatic blue, orange and red light. (A) Changes in PSI:PSII fluorescence emission ratio during acclimation to blue (diamonds), orange (triangles) and red light (circles). The PSI:PSII ratio was estimated from 77 K fluorescence emitted by PSI (725 nm; F_725_) relative to PSII (695 nm; F_695_) after excitation of chlorophyll *a* at 440 nm. (B) Changes in the ratio of PBS excitonically coupled to PSI vs PBS coupled to PSII, estimated from 77 K fluorescence emitted by PSI relative to PSII after excitation of phycocyanin at 590 nm. (C) Changes in the ratio of uncoupled phycocyanin vs PBS coupled to PSII, estimated from 77 K fluorescence emitted by phycocyanin (650 nm; F_650_) relative to PSII after excitation of phycocyanin at 590 nm. Red‐dotted squares in (A‐C) indicate steady‐state fluorescence ratios of the dilute chemostat in red light. Data are averages of three biological replicates ± SD (n = 3). The 77 K fluorescence emission spectra are shown in Fig. S1.

Low‐temperature fluorescence spectra of cells flash‐frozen at 77 K offer an estimate of the relative abundances of the two photosystems and their association with PBS. The PBS and photosystems in these frozen cells have a fixed position, but still absorb photons and transfer the excitation energy to the reaction centers. Upon excitation of Chl *a* at 440 nm, fluorescence is emitted by PSII at 695 nm (F_695_) and by PSI at 725 nm (F_725_) (Fig. S1). After the switch to orange and red light, the PSI:PSII fluorescence emission ratio remained rather constant around 2.55 ± 0.34 (Fig. 2A), which is within the typical range for *Synechocystis* sp. PCC 6803 and other cyanobacteria (Shen et al. 1993, Murakami et al. 1997, Kirilovsky 2015). In contrast, in blue light the PSI:PSII fluorescence emission ratio decreased drastically to 0.90 ± 0.01 in steady‐state. Cells in the dilute chemostat in red light showed a similar PSI:PSII fluorescence emission ratio as those in the orange and red light chemostats, indicating that the low PSI:PSII ratio in blue light is a response to light color rather than to light intensity (Fig. 2A).

Upon excitation of PBS at 590 nm, fluorescence is emitted by PSII coupled to PBS (F_695_), by PSI coupled to PBS (F_725_) and by PBS not involved in light transfer to any of the two photosystems (uncoupled PBS at 650 nm; F_650_) (Fig. S1). The fluorescence emission peaks indicate that the distribution of PBS over the two photosystems was not affected by the switch from artificial white light to monochromatic orange and red light (Fig. 2B). In contrast, the switch to blue light strongly decreased the coupling of PBS to PSI relative to PSII (Fig. 2B) and increased the amount of uncoupled PBS (Fig. 2C). The distribution of PBS in the dilute chemostat in red light showed a qualitatively similar but less pronounced response as in blue light (Fig. 2B,C).

### Blue light has more effect on the transcriptome than orange and red light

Microarrays were used to study changes in gene expression of *Synechocystis* sp. PCC 6803 after the switch from artificial white light to monochromatic blue, orange and red light. We calculated log_2_ fold changes in gene expression, by comparing the transcriptome at time points after the light switch with the transcriptome before the light switch (i.e. at t = 0 h). The complete transcriptome data are presented in Table S2, and the regulated genes are detailed in Table S3. In total, 145 of the 3264 investigated genes were significantly up‐ or downregulated at one or more time points in blue light. In contrast, only five genes were differentially expressed in orange light, four genes in red light, and two genes in the dilute chemostat in red light. Hence, the switch to blue light induced a much larger transcriptome response than the switch to orange and red light.

**Figure 3:**
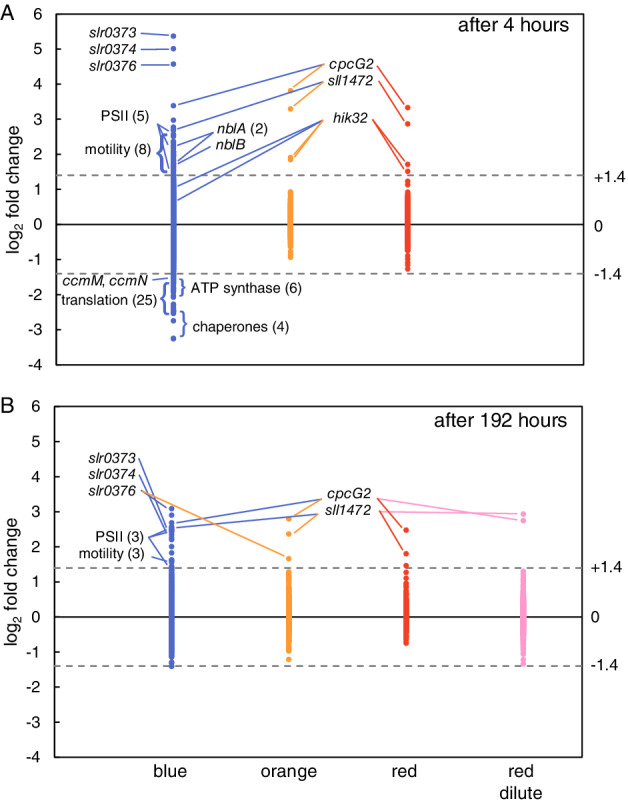
Overview of changes in gene expression of *Synechocystis* sp. PCC 6803, after a switch from artificial white light to monochromatic blue, orange and red light. (A) Gene expression levels of all analyzed genes, 4 h after the switch to blue (left), orange (middle) and red light (right). (B) Gene expression levels during steady state of the chemostats, 192 h after the switch to blue (left), orange (middle) and red light (right). Pink data points on the far right indicate gene expression by cells in the dilute chemostat in red light. Gene expression was compared to the expression levels in steady‐state cells acclimated to artificial white light at t = 0 h. Dashed horizontal lines indicate the threshold log_2_ fold changes for significantly upregulated (> + 1.4) and downregulated (<−1.4) genes. Names or functional descriptions of the strongly regulated (groups of) genes are indicated. Bracketed values indicate the number of regulated genes in a functional group. The data points cover all 3264 genes investigated in this study, and are based on three biological replicates (n = 3) for each light color. A complete list of all differentially expressed genes can be found in Table S3.

Many of the observed changes in gene expression already occurred within the first 4 h after the light switch (Fig. 3A). After 4 h in orange and red light only four genes, which all belong to the light‐sensing *ccaS* gene cluster, were significantly upregulated (i.e., with a log_2_ fold change >1.4), while none of the investigated genes were downregulated. In contrast, after 4 h in blue light, 115 genes were differentially expressed, of which 61 genes were upregulated and 55 were downregulated (Fig. 3A).

After 192 h, when the chemostats had reached a new steady‐state, only three genes were still upregulated in orange and red light, whereas 21 genes were upregulated and one was downregulated in blue light (Fig. 3B). Moreover, only two genes were upregulated in the dilute chemostat in red light. These results imply that most observed changes in gene expression are a response to blue light specifically rather than to light intensity, and that many of these responses turn out to be transient.

### Response of genes related to photosynthesis and respiration

**Figure 4:**
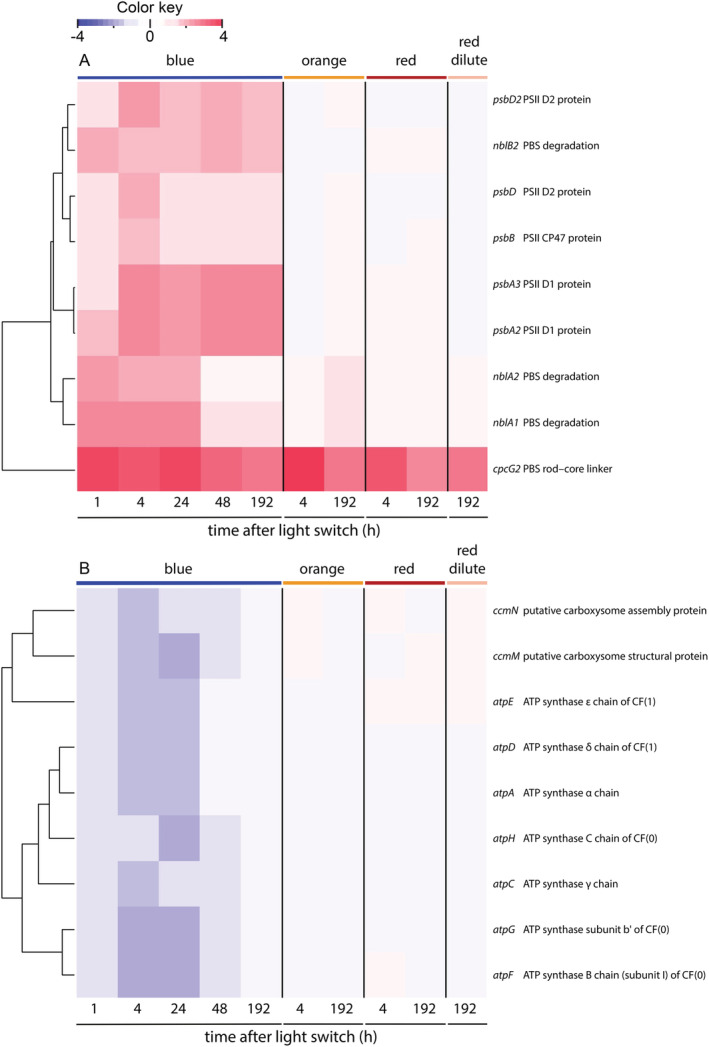
Expression of genes related to photosynthesis and respiration of *Synechocystis* sp. PCC 6803, after a switch from artificial white light to monochromatic blue, orange and red light. Changes in expression of genes related to (A) PSII and PBS, and (B) carboxysome production and ATP synthase, at several time points after the switch to monochromatic blue, orange and red light. Genes related to PSI, cytochrome *b*_*6*_ *f*, NADH turnover, respiratory terminal oxidases and soluble electron carriers were not differentially expressed in any of the experimental light conditions. Gene expression after the switch was compared to expression of cells acclimated to white light prior to the switch (i.e. at t = 0 h). Changes in gene expression in the dilute chemostat in red light are also indicated. Expression is shown only for genes that were significantly regulated (*P* < 0.05) and displayed a log_2_ fold change <−1.4 or >+1.4 for at least one of the time points. A complete list of all differentially expressed genes can be found in Table S3. The heatmaps are ordered based on hierarchical clustering of the gene expression patterns. The data are based on three biological replicates (n = 3) for each light color.

We used Cyanobase (http://genome.kazusa.or.jp/cyanobase) as a guideline to distinguish between genes of different functional categories. Furthermore, we have added the annotation of several genes that are listed as unknown or hypothetical in CyanoBase, but are annotated in the Kyoto Encyclopedia of Genes and Genomes (KEGG) (https://www.kegg.jp/), or in recent literature. Annotations of all differentially expressed genes can be found in Table S3. Several of the significantly regulated genes were in the category photosynthesis and respiration (Fig. 4). In blue light, five genes related to PSII (*psbA2*, *psbA3*, *psbD*, *psbD2*, *psbB*), encoding two D1 proteins, two D2 proteins and a CP47 protein, respectively, were strongly upregulated (Fig. 4A). Furthermore, four genes related to PBS, encoding the PBS degradation proteins NblA1, NblA2 and NblB2 and the rod‐core linker protein CpcG2, were also upregulated. Conversely, seven genes (*atpA*, *atpC‐atpH*) encoding structural proteins of the adenosine triphosphate (ATP) synthase complex, and two genes (*ccmM*, *ccmN*) encoding carboxysome proteins, were significantly downregulated in blue light (Fig. 4B).

In orange and red light and in the dilute chemostat in red light, the only gene within the category of photosynthesis and respiration that displayed a significant change in expression level was *sll1471* encoding the PBS rod‐core linker protein CpcG2 (Fig. 4A). This gene was also upregulated in blue light, and belongs to the *ccaS* gene cluster (Hirose et al. 2008).

Genes related to PSI, cytochrome *b*_*6*_ *f*, NADH turnover, respiratory terminal oxidases and soluble electron carriers were not differentially expressed in any of the tested conditions; in addition, neither were any of the genes encoding photoprotective proteins, such as the orange carotenoid protein, iron‐stress inducible protein A or flavodiiron proteins.

### Genes related to other functional categories

**Figure 5:**
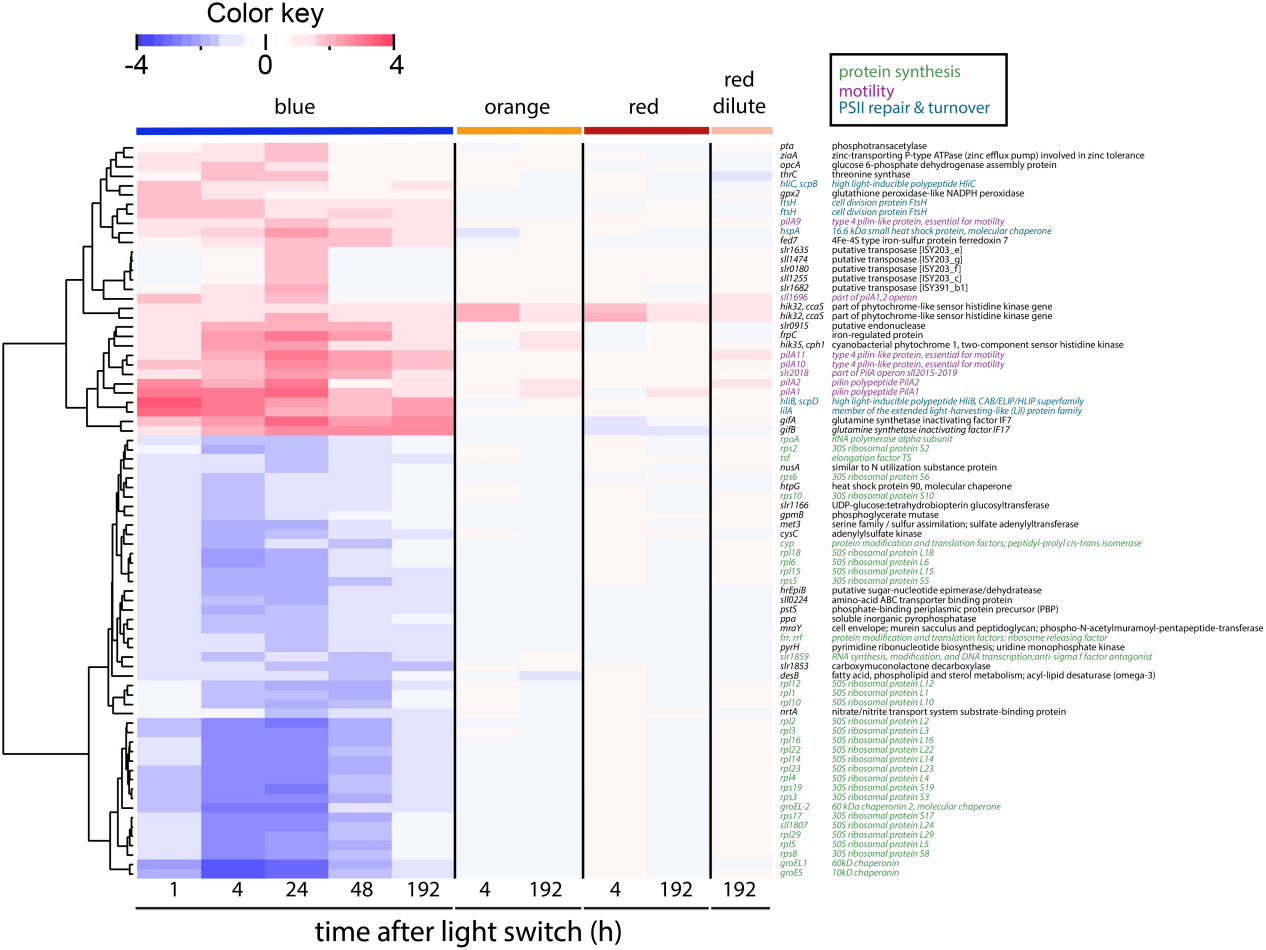
Expression of genes from several functional categories of *Synechocystis* sp. PCC 6803, after a switch from artificial white light to monochromatic blue, orange and red light. The heatmap shows changes in expression for all known genes that were regulated in response to the light switch, except those related to photosynthesis and respiration (which are shown in Fig. 4). Genes encoding proteins involved in protein synthesis and stability (green), motility (magenta), PSII repair and turnover (light blue) as annotated in CyanoBase, KEGG, or recent literature, are highlighted in color. Genes classified as hypothetical or unknown genes are shown in Fig. S2. The layout and analyses are as in Fig. 4.

In the other functional categories, again many more genes were differentially expressed in blue light than in orange and red light (Fig. 5). Specifically, 29 genes belonging to other known functional categories were significantly upregulated in blue light, including genes encoding proteins involved in cellular motility (7 genes), PSII repair and turnover (6 genes), transposases (5 genes), histidine kinases (*hik32* and *hik35*) and the glutamine synthetase inactivating factors *gifA* and *gifB*. Conversely, 47 genes of the other functional categories were significantly downregulated in blue light, including genes encoding ribosomal proteins (24 genes), other proteins involved in transcription and translation (6 genes), chaperones (4 genes) and proteins involved in amino acid biosynthesis (2 genes).

The only two genes in the other functional categories that were differentially expressed in orange and red light and in the dilute chemostat in red light encode the histidine kinase Hik32, interrupted by an insertion sequence (Okamoto et al. 1999) (Fig. 5; Table S3). *Hik32* also belongs to the previously mentioned *ccaS* gene cluster (Hirose et al. 2008).

In the categories of hypothetical genes and genes with unknown function, 40 genes were upregulated and 10 genes were downregulated in blue light (Fig. S2). The three most strongly upregulated genes in blue light (*slr0373*, *slr0374* and *slr0376*; see also Fig. 3A) are hypothetical genes that are located in a single stress‐related gene operon (Singh and Sherman 2002). The gene *sll1472* was upregulated in all three light colors, and is again part of the *ccaS* gene cluster (Hirose et al. 2008).

## Discussion

### Blue light alters photophysiology

Our results show that *Synechocystis* sp. PCC 6803 produces much less biomass in blue light than in orange, red and artificial white light (Fig. 1). The low biomass production of PBS‐containing cyanobacteria in blue light is consistent with many previous findings (Wyman and Fay 1986, Wilde et al. 1997, Wang et al. 2007, Singh et al. 2009, Chen et al. 2010, Choi et al. 2013, Bland and Angenent 2016, Luimstra et al. 2018). Blue light at wavelengths ≤450 nm is not absorbed by the PBS (Tandeau de Marsac 2003, Six et al. 2007), which usually transfer most of their absorbed light energy to PSII. Blue light is absorbed by Chl *a* and carotenoids. Cyanobacteria tend to invest much more of their Chl *a* in PSI than in PSII (Myers et al. 1980, Fujita 1997). Moreover, in cyanobacteria, only carotenoids of PSI appear to be involved in light harvesting, whereas carotenoids of PSII are involved in heat dissipation (Stamatakis et al. 2014). Hence, the low photosynthetic efficiency of cyanobacteria in blue light is commonly explained by the hypothesis that blue light causes an excitation imbalance between the two photosystems, with more light absorption by PSI than by PSII (Fujita 1997, Solhaug et al. 2014, Kirilovsky 2015, Luimstra et al. 2019). This hypothesis also provides a rationale for the strong decrease of the PSI:PSII fluorescence emission ratio (Fig. 2A; Fig. S1A) and the decreased coupling of PBS to PSI, relative to PSII, after the switch from white to blue light (Fig. 2B; Fig. S1D). Similar physiological changes in response to blue light have been observed in previous studies (Tsinoremas et al. 1994, Wilde et al. 1997, El Bissati and Kirilovsky 2001, Singh et al. 2009, Luimstra et al. 2018), and reflect increased investments in PSII and/or decreased investments in PSI to compensate for the excitation imbalance between the two photosystems.

The strong physiological response to blue light is also reflected in the transcriptome data, which showed that 145 genes responded to a switch from white to blue light whereas only a handful of genes responded to orange or red light (Fig. 3). In blue light several genes encoding structural PSII proteins were upregulated (Fig. 4A), indicative of an effort to overcome the low amount of excitons reaching PSII relative to PSI. This response included an increased expression of the D1 and D2 proteins that form the core of the PSII reaction center, and the PSII core antenna protein CP47. Furthermore, we observed an increase in several genes related to PSII turnover, including 2 *ftsH* genes, *hliB*, *hliC* and *lilA* (Fig. 5). The *ftsH* genes encode proteins that are involved in the degradation of damaged D1 (Cheregi et al. 2007) and quality control of newly synthesized D1 (Komenda et al. 2006). The *hliB* and *hliC* genes encode high‐light inducible proteins that are suggested to stabilize PSII subunits during PSII repair (Promnares et al. 2006, Yao et al. 2007). The *lilA* gene encodes a protein of the light‐harvesting protein family that associates with HliB and HliC (Kufryk et al. 2008). In contrast to this strong response of several genes related to PSII synthesis, none of the genes encoding PSI subunits were differentially expressed in blue light. Hence, the observed decrease in the PSI:PSII fluorescence emission ratio in blue light (Fig. 2A; Fig. S1A) seems to originate from an increased expression of PSII rather than a decreased expression of PSI. This is consistent with previous research using *Synechocystis* sp. PCC 6803, where blue light resulted in an upregulation of *psbA* encoding the PSII D1 protein, while expression levels of *psaE* encoding the Psa‐E subunit of PSI remained similar (El Bissati and Kirilovsky 2001).

Furthermore, in blue light we observed an increase in the expression of *nblA1* and *nblA2*, encoding two PBS degradation proteins (Fig. 4A). The gene *slr1687* is presumed to encode another PBS degradation protein, NblB2 (Li and Sherman 2002), and was also upregulated in blue light (Fig. 4A). Moreover, the expression of a molecular chaperone that is involved in stabilization of PBS (HtpG, see Sato et al. 2010) was downregulated (Fig. 5). Degradation of PBS might be induced because these antennae are not functional in blue light. Nitrogen‐rich PBS can constitute up to 50% of the total cellular protein content in cyanobacteria (Grossman et al. 1993), and their degradation provides building blocks for the production of other proteins. Indeed, the 77 K fluorescence spectra showed an increased decoupling of PBS from the photosystems in blue light (Fig. 2C; Fig. S1D). However, whole‐cell absorption spectra still showed considerable light absorption by phycocyanin at 625 nm relative to Chl *a* (Fig. 1C), while previous research has shown that PBS degradation induced by NblA1 and NblA2 is accompanied by a decreased absorption by phycocyanin (Baier et al. 2001). Hence, although changes in gene expression appear to prepare the degradation of PBS and disassembly of the PBS may have been initiated, the degradation process is not yet fully activated in nutrient‐replete cells exposed to blue light.

### Blue light induces flocculation and pili formation

Another interesting observation is the blue‐light induced upregulation of genes that play a role in phototactic motility (Fig. 5). In particular, our results show that the switch to blue light resulted in initial upregulation of *pilA1*, known to be essential for the formation of Type‐IV pili and hence for twitching motility (Bhaya et al. 2000), and of *pilA2* and *sll1696* which are part of the same operon. Type IV‐based motility facilitates gliding of the cells over short distances on moist surfaces (Bhaya et al. 2001). Previous studies have indeed shown that blue light increases motility and negative phototaxis of *Synechocystis* sp. PCC 6803 (Ng et al. 2003, Terauchi and Ohmori 2004, Fiedler et al. 2005).

After initial upregulation of the *pilA1*‐*pilA2* operon, the *pilA9*, *pilA10* and *pilA11*‐containing operon *slr2015*‐*slr2019* (encoding minor pili subunits) was also upregulated in blue light (Fig. 5). PilA9‐PilA11 have recently been shown to modulate the adhesive properties of Type‐IV pili and to enhance flocculation of the cells (Yoshihara and Ikeuchi 2004, Panichkin et al. 2006, Conradi et al. 2019). This was consistent with our observations, which showed increased flocculation and wall growth in the blue‐light chemostats (V.M. Luimstra, personal observation), a phenomenon that has also been described in previous studies (Enomoto et al. 2015, Agostoni et al. 2016). Flocculation and negative phototaxis may be interpreted as a stress response, protecting *Synechocystis* cells from adverse light conditions imposed by blue light. Moreover, in *Synechocystis* sp. PCC 6803, type‐IV pili are essential for the uptake of extracellular DNA (Yoshihara et al. 2001) and flocculation of the cells might therefore also contribute to horizontal gene transfer (Conradi et al. 2019).

### Transient changes in gene expression related to reduced growth

Remarkably, although genes associated with PSII and motility were upregulated in response to blue light for the entire duration of the experiment, many other genes were differentially expressed only temporarily after the switch to blue light and thereafter returned to their initial expression levels (Figs. 4B, 5; Fig. S2). These transient changes in gene expression can be attributed to a temporary reduction of the growth rate after the switch to blue light, as illustrated by the declining cell numbers and biomass (Fig. 1A,B). At steady state, the growth rate equals the dilution rate of the chemostat. Hence, after ~150 h, the cell numbers stabilized at a new steady state and the growth rate recovered to the same value as before the light switch.

Low growth rates are usually associated with low ribosomal abundances (Scott et al. 2014, Zavřel et al. 2019). The transient period of low growth rate in blue light was indeed accompanied by a temporary down‐regulation of 24 genes encoding ribosomal proteins and several other genes involved in transcription and translation (Fig. 5). Cellular metabolism is strongly ATP‐dependent, which explains why genes encoding ATP‐synthase were also temporarily downregulated during the transient period of reduced growth (Fig. 4B). Furthermore, several genes encoding chaperones (*groES*, *groEL1*, *groEL2* and *htpG*), which commonly play an important role in the folding and stabilization of proteins, were also temporarily downregulated in blue light (Fig. 5). Interestingly, the only chaperone that was temporarily upregulated in blue light was the small heat‐shock protein HspA (Fig. 5), which plays a role in the stabilization of PSII and the thylakoid membrane (Nitta et al. 2005, Sakthivel et al. 2009).

### Light sensing in cyanobacteria

To allow acclimation to changes in their environment, microorganisms have developed highly efficient mechanisms to sense, transduce and respond to external signals. Cyanobacteria often use two‐component systems composed of a histidine kinase (Hik) and a cognate response regulator (Rre) (Appleby et al. 1996, Mizuno et al. 1996, Capra and Laub 2012). In total, *Synechocystis* contains 49 Hiks, of which six Hiks that have phytochrome‐like features are suggested to function as light sensors (hik1, hik3, hik24, hik32, hik35 and hik44; see Xu and Wang 2019). Of these six Hiks, only *hik35* and *hik32* were differentially expressed in our experiments.

*Hik35* (*slr0473*) encodes the cyanobacterial phytochrome‐like protein Cph1 (Hughes et al. 1997) and appears to be involved in the regulation of at least 10 genes (Hübschmann et al. 2005). *Hik35* and five Hik35‐regulated genes (*gifA*, *thrC*, *lilA*, *slr0373*, *slr0376*) were upregulated in our experiments after the switch to blue light, whereas one of the Hik35‐regulated genes (*tsf*) was downregulated. *Tsf* encodes the elongation factor TS which is involved in translation. The Hik35‐regulated genes *slr0373* and *slr0376* form an operon with *slr0374*, and together these three genes represent the most strongly regulated genes in blue light (Fig. 3). The function of this operon is yet unknown, but it is known to respond to different stress conditions, such as iron deficiency, sulfur deficiency, nitrogen deficiency, high salinity, high light and oxidative stress (Singh and Sherman 2002, Singh et al. 2004). The *slr0374* gene encodes the highly conserved protein Ycf46. Inactivation of this gene reduces activity of the extracellular carbonic anhydrase EcaB, which indicates that it plays a role in the regulation of photosynthetic carbon fixation (Jiang et al. 2015).

Hik32, also known as CcaS, is encoded by two *hik32* genes (*sll1473* and *sll1475*; see Kondo et al. 2007), which were among the few genes that were significantly upregulated in orange and red light (Fig. 3). These two *hik32* genes are interrupted by a transposase (*sll1474*) in the *Synechocystis* strain (‘Kazusa’) sequenced in Cyanobase, but have been shown to form one gene in the original PCC 6803 strain that was used here (Okamoto et al. 1999). The two‐component system histidine kinase Hik32 and the cognate upstream response regulator CcaR (*slr1584*) regulate the expression of *cpcG2* (*sll1471*) and the hypothetical gene *sll1472* (Hirose et al. 2008), which were the two most strongly upregulated genes in orange and red light (Fig. 3). *CpcG2* encodes a rod‐core linker protein that leads to the formation of atypical PBS that lack the allophycocyanin core and preferentially transfer energy to PSI, in contrast to the conventional PBS (containing CpcG1) that mainly transfer light energy to PSII (Kondo et al. 2005, 2007). The phycocyanin‐containing PBS of *Synechocystis* sp. PCC 6803 absorb orange and red light very effectively (Tandeau de Marsac 2003), and usually transfer most of the absorbed light energy to PSII. Hence, the strong upregulation of *cpcG2* in orange and red light might be interpreted as a functional adaptation to redistribute part of the light energy absorbed by PBS to PSI, presumably to maintain the excitation balance between PSI and PSII in orange and red light. It remains to be elucidated why *cpcG2* and the hypothetical gene *sll1472* were also upregulated after the transfer from white to blue light (Fig. 4; Fig. S2). Possibly, they might play a role in the observed uncoupling of PBS from the photosystems in blue light (Fig. 2C; Fig. S1D).

None of the additional (blue light) photoreceptors (Fiedler et al. 2005, Moon et al. 2010) showed a measurable transcriptional response to the imposed changes in light conditions.

### Comparison with other studies

To the best of our knowledge, only one study has thus far focused on changes in the transcriptome of cyanobacteria in response to blue light (Singh et al. 2009). In their experiments, cells from a relatively dense *Synechocystis* culture acclimated to white light were inoculated in dilute batch cultures that were exposed to either blue or red light. Gene expression levels were measured only during the first 6 h of their experiments. Singh et al. (2009) observed a similar physiological response of *Synechocystis* sp. PCC 6803 as in our study, with a lower PSI:PSII ratio in blue light compared to red or white light. Some cautionary notes should be made before comparing their gene expression data with our results, however. In particular, Singh et al. (2009) quantified gene expression as the log_2_ ratio of gene expression levels in red light relative to expression levels in blue light. Therefore, if gene expression was designated as, e.g., higher in red light it is uncertain whether this response actually reflected upregulation in red light, downregulation in blue light, or both. Furthermore, their batch cultures in red light showed a strong increase in cell numbers, while cell numbers in blue light barely increased during the first ~24 h. Hence, it is likely that differences in gene expression between red and blue light observed in their experiments reflect differences in light color, average light intensity perceived by the cells, and growth rate. This contrasts with our experiments, where we have used chemostats to separate transient effects caused by temporary changes in growth rate from persistent effects caused by differences in light color. Furthermore, we have used a dilute chemostat to separate effects of light color and light intensity.

Despite these differences in experimental design, Singh et al. (2009) also report major changes in the expression of photosynthesis genes, with a higher expression of genes associated with PSII (and its synthesis) in blue light than in red light, including *hliB*, *hliC*, *lilA* and *hspA*, which are involved in PSII turnover and protection. Their Table S2 indicates that they also observed that the putative pilin genes *pilA9‐pilA11* (related to motility) and the stress‐response operon containing *slr0373*, *slr0374* and *slr0376* had higher expression levels in blue light than in red light. Furthermore, in accordance with the lower growth rate in blue light, they also observed lower transcript levels of genes encoding many ribosomal proteins, ATP synthase subunits and the chaperones GroES, GroEL1, GroEL2 after 3–6 h in blue light.

In contrast to our findings, however, Singh et al. (2009) also observed a lower expression of several PSI genes in blue light than in red light. Furthermore, they report that expression of the motility genes *pilA1* and *pilA2*, and the PBS degradation genes *nblaA1* and *nblA2* was higher in red light than in blue light. This might be related to the lower light intensities used in their experiments, in line with results of Ogawa et al. (2018). Also, an increased expression of the *cpcG1* gene encoding the conventional rod‐core linker protein of the PBS was observed in blue light while no data for the alternative *cpcG2* gene were available from their experiments. In addition, contrary to our results, Singh et al. (2009) found large differences in cellular carbon and nitrogen metabolism between red and blue light. This might be related to differences in growth conditions between red and blue light in the experiments of Singh et al. (2009), where cells in blue light were still in initial lag phase and had not yet resumed growth.

Hübschmann et al. (2005) investigated differences in gene expression between batch cultures of *Synechocystis* sp. PCC 6803 exposed to red light (652 nm) and far‐red light (734 nm). Red light of 652 nm is absorbed by Chl *a* and can also be absorbed by allophycocyanin in the core of the PBS, whereas far‐red light is absorbed only by Chl *a* and not by PBS (Lemasson et al. 1973, Glazer and Bryant 1975, MacColl 2004). Hence, we have theorized previously (Luimstra et al. 2018) that, similar to blue light, growth in far‐red light may also lead to an excitation imbalance between PSI and PSII. In line with this prediction, Murakami et al. (1997) showed that red light of 680 nm (which is not absorbed by PBS) resulted in a substantially lower PSI:PSII ratio than red light of 650 nm. Hübschmann et al. (2005) found that far‐red light of 734 nm indeed resulted in lower growth rates than red light of 652 nm. Moreover, many genes that were up‐regulated in blue light in our experiments were similarly up‐regulated in far‐red light in their experiments, including several genes related to PSII synthesis (*psbA2*, *psbA3*, *psbD2*, *psbB*) and to PSII stabilization and turnover (*hliB*, *hliC*, *lilA*, *hspA*). Similarly, their Table S2 shows that the PBS degradation genes *nblA2* and *nlbB2*, and the genes *slr0374* and *slr0376* from the stress‐responsive operon described by Singh and Sherman (2002) were also upregulated in far‐red light. Furthermore, Hübschmann et al. (2005) found that the expression of more than 20 genes related to ribosomal proteins and several genes related to ATP synthase was lower in far‐red light than in red light, in line with the transient changes in gene expression when blue light temporarily reduced the growth rate in our experiments. This comparison indicates that many genes involved in photosynthesis and growth are similarly regulated in blue and in far‐red light, suggesting that these genes are indeed affected by the excitation imbalance between the two photosystems.

However, some genes that were strongly upregulated or downregulated in blue light were not differentially expressed in far‐red light, indicating that these genes responded to blue light specifically. For example, *hik35* encoding the cyanobacterial phytochrome Cph1 was upregulated in blue light in our study, but was not differentially expressed in red or far‐red light in the experiments of Hübschmann et al. (2005). And while blue light downregulated the expression of genes encoding the chaperones GroES, GroEL2 and HtpG in our experiments, these genes were upregulated in far‐red light in their experiments. Hübschmann et al. (2005) also found that most pilin genes were not regulated or even downregulated in far‐red light. By contrast, in our experiments blue light induced upregulation of several pilin‐genes involved in motility and flocculation of cells (Fig. 5), in line with other research showing that blue light has distinct effects on cellular motility and flocculation (Ng et al. 2003, Terauchi and Ohmori 2004, Fiedler et al. 2005, Savakis et al. 2012, Enomoto et al. 2015, Agostoni et al. 2016).

## Conclusions

Our results show that the switch from artificial white to blue light had a much stronger effect on the gene expression profile, photophysiology and growth of *Synechocystis* sp. PCC 6803 than the switch to orange and to red light. In particular, PSII genes were upregulated in blue light, in agreement with the decreased PSI:PSII ratio of the cells. This photophysiological acclimation will improve the distribution of excitation energy between PSII and PSI, even though the low growth rate in blue light indicates that upregulation of PSII was insufficient to fully restore linear photosynthetic electron flow. Conversely, many ribosomal genes and other genes involved in protein synthesis were temporarily downregulated in blue light, concomitant with the transient decline of the growth rate. Hence, blue light not only results in a limited transfer of excitation energy to PSII and a low photosynthetic efficiency of PBS‐containing cyanobacteria, but also induces marked changes in their transcriptome to counter these adverse light conditions.

## Author contributions

All authors were involved in designing the experiments. V.M.L. performed the experiments with technical support from J.M.S. J.M.S. and V.M.L. analyzed the microarray data. V.M.L. and J.H. wrote the manuscript and all authors except our late colleague H.C.P.M commented on the final version.

*Acknowledgements* – We are most grateful to Selina van Leeuwen (Microarray Department, University of Amsterdam) for her advice and technical support of the microarray analysis, and we thank the two reviewers for their helpful comments. This work has been performed in the cooperation framework of Wetsus, European Centre of Excellence for Sustainable Water Technology (http://www.wetsus.eu). Wetsus is co‐funded by the Dutch Ministry of Economic Affairs and Ministry of Infrastructure and Environment, the Province of Fryslân and the Northern Netherlands Provinces.

## Supporting information


**Fig. S1.** Low‐temperature (77 K) fluorescence emission spectra of *Synechocystis* sp. PCC 6803, before and after a switch from artificial white light to monochromatic blue, orange and red light.

**Fig. S2.** Expression of ‘hypothetical’ and ‘unknown’ genes of *Synechocystis* sp. PCC 6803, after a switch from artificial white light to monochromatic blue, orange and red light.

**Table S1.** Loop design used to hybridize samples to the microarray chips.

**Table S2.** Complete transcriptome data of this study.

**Table S3.** Genes that were differentially expressed during the study and their annotation.

## Data Availability

The data that support the findings of this study are available in the Supporting Information of this article.
