# Supplementary material for: Blue light induces major changes in the gene expression profile of the cyanobacterium Synechocystis sp. PCC 6803
Source: Physiol Plant. 2020 Mar 14;170(1):10–26. doi: 10.1111/ppl.13086 (PMC7496141; doi:10.1111/ppl.13086)
Supplement: Supplementary file 1 — Fig. S1. Low‐temperature (77 K) fluorescence emission spectra of Synechocystis sp. PCC 6803, before and after a switch from artificial white light to monochromatic blue, orange and red light. [file PPL-170-10-s001.pdf]

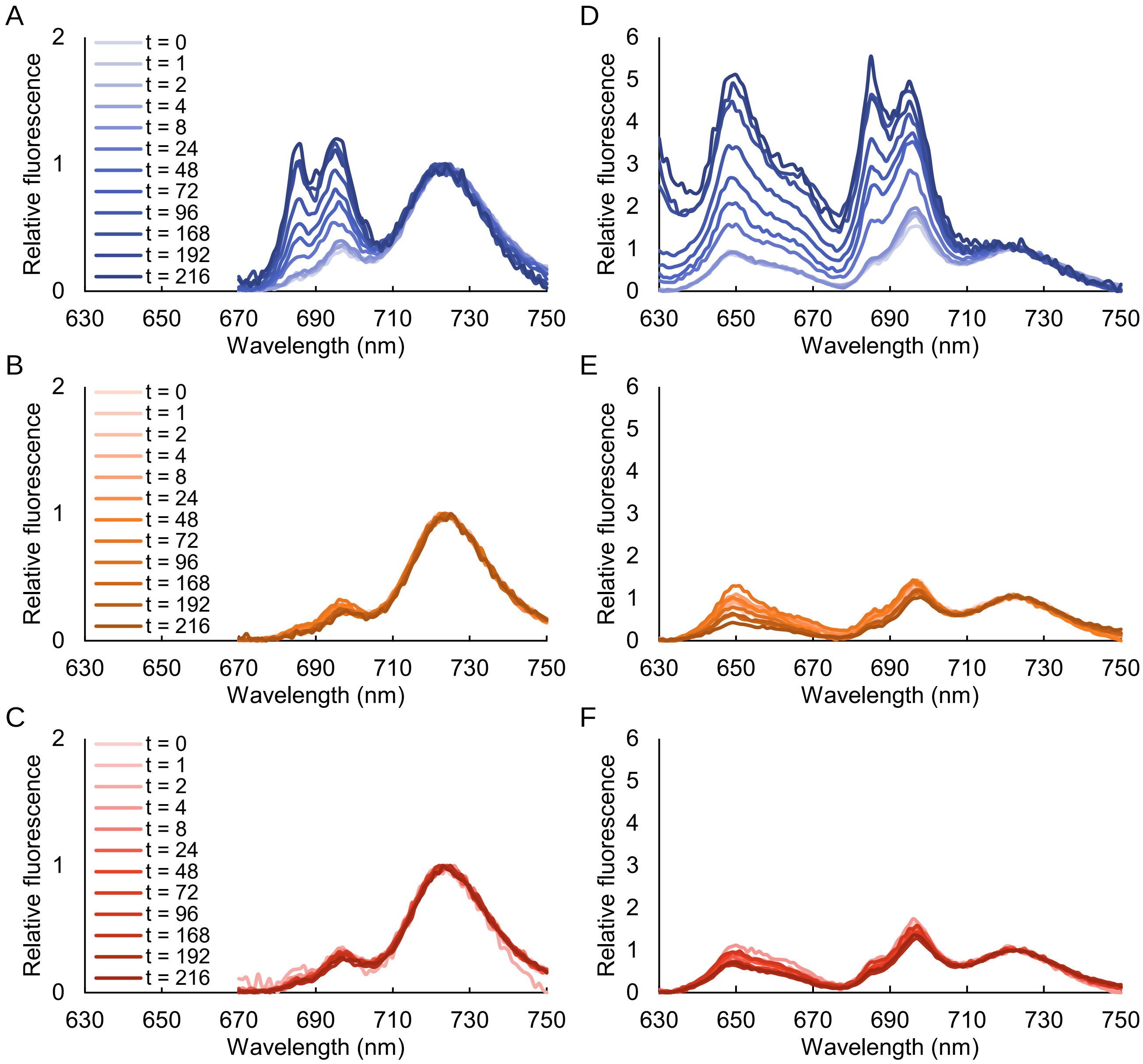

Supplemental Fig. S1. Low-temperature (77 K) fluorescence emission spectra of *Synechocystis* sp. PCC 6803, before and after a switch from artificial white light to monochromatic blue, orange and red light. 77 K spectra were measured before (t=0 h) and at several time points after the switch to (A,D) blue light, (B,E) orange light, and (C,F) red light. (A-C) Excitation of Chl a at 440 nm yields fluorescence emission peaks at 695 nm for PSII and at 725 nm for PSI. (D-F) Excitation of phycocyanin at 590 nm yields fluorescence emission peaks at 695 nm when PBSs are excitonically coupled to PSII and at 725 nm when coupled to PSI (state 2). PBS that are decoupled from the photosystems and phycocyanin that is not incorporated into PBSs result in fluorescence emission from phycocyanin and allophycocyanin at 650-665 nm. Spectra show the averages of three biological replicates per time point (n=3), and are normalized to PSI fluorescence at 725 nm.
