# Supplementary material for: Blue light induces major changes in the gene expression profile of the cyanobacterium Synechocystis sp. PCC 6803
Source: Physiol Plant. 2020 Mar 14;170(1):10–26. doi: 10.1111/ppl.13086 (PMC7496141; doi:10.1111/ppl.13086)
Supplement: Supplementary file 2 — Fig. S2. Expression of ‘hypothetical’ and ‘unknown’ genes of Synechocystis sp. PCC 6803, after a switch from artificial white light to monochromatic blue, orange and red light. [file PPL-170-10-s002.pdf]

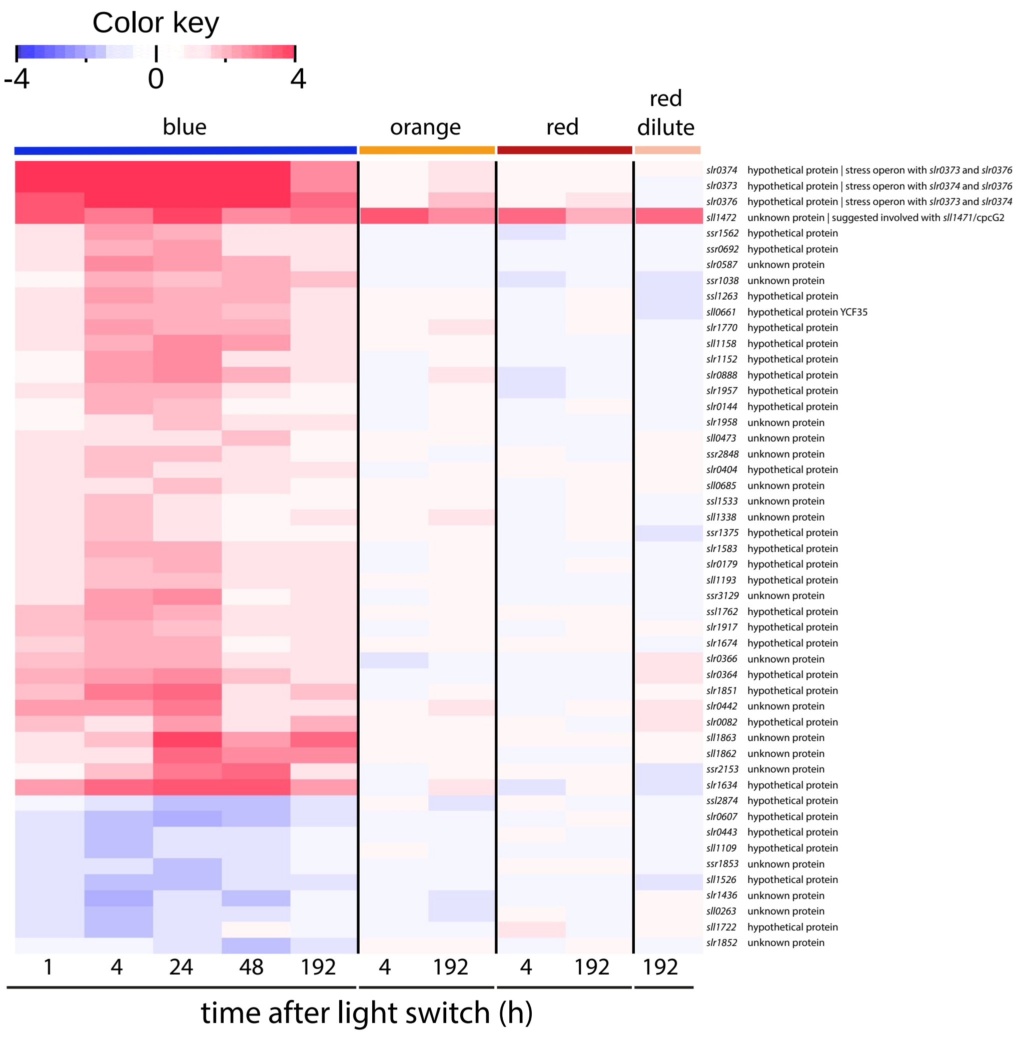

Supplementary Fig. S2. Expression of 'hypothetical' and 'unknown' genes of *Synechocystis* sp. PCC 6803, after a switch from artificial white light to monochromatic blue, orange and red light. The heatmap shows changes in expression for all genes classified as hypothetical and unknown genes that were regulated in response to the light switch. The layout and analyses are as in Figs. 4 and 5.
