# Supplementary material for: Blue light induces major changes in the gene expression profile of the cyanobacterium Synechocystis sp. PCC 6803
Source: Physiol Plant. 2020 Mar 14;170(1):10–26. doi: 10.1111/ppl.13086 (PMC7496141; doi:10.1111/ppl.13086)
Supplement: Supplementary file 3 — Table S1. Loop design used to hybridize samples to the microarray chips. [file PPL-170-10-s003.pdf]

Table S1: Loop design used to hybridize samples to the microarray chips.

Color	Sample	Replicate	Array	Dye	Chip
‘white'	t = 0 h	1	1	Cy3	1
‘white'	t = 0 h	2	10	Cy5	1
‘white'	t = 0 h	3	11	Cy3	1
‘white'	t = 0 h	4	12	Cy5	1
‘white'	t = 0 h	5	13	Cy3	1
blue	t = 1 h	1	1	Cy5	1
blue	t = 1 h	2	2	Cy3	1
blue	t = 1 h	3	15	Cy5	1
blue	t = 4 h	1	2	Cy5	1
blue	t = 4 h	2	3	Cy3	1
blue	t = 4 h	3	16	Cy5	1
blue	t = 8 h	1	3	Cy5	1
blue	t = 8 h	2	4	Cy3	1
blue	t = 8 h	3	14	Cy5	1
blue	t = 24 h	1	4	Cy5	1
blue	t = 24 h	2	5	Cy3	1
blue	t = 24 h	3	15	Cy3	1
blue	t = 192 h	1	5	Cy5	1
blue	t = 192 h	2	6	Cy3	1
blue	t = 192 h	3	11	Cy5	1
orange	t = 4 h	1	9	Cy5	1
orange	t = 4 h	2	10	Cy3	1
orange	t = 4 h	3	14	Cy3	1
orange	t = 192 h	1	6	Cy5	1
orange	t = 192 h	2	7	Cy3	1
orange	t = 192 h	3	13	Cy5	1
red	t = 4 h	1	8	Cy5	1
red	t = 4 h	2	9	Cy3	1
red	t = 4 h	3	16	Cy3	1
red	t = 192 h	1	7	Cy5	1
red	t = 192 h	2	8	Cy3	1
red	t = 192 h	3	12	Cy3	1
red	t = 192 h	1	3	Cy5	2
red	t = 192 h	2	4	Cy3	2
red	t = 192 h	3	6	Cy3	2
red	red dilute t = 192 h	1	4	Cy5	2
red	red dilute t = 192 h	2	5	Cy3	2
red	red dilute t = 192 h	3	7	Cy3	2
blue	t = 48 h	1	1	Cy5	2
blue	t = 48 h	2	2	Cy3	2
blue	t = 48 h	3	7	Cy5	2
blue	t = 192 h	1	2	Cy5	2
blue	t = 192 h	2	3	Cy3	2
blue	t = 192 h	3	8	Cy3	2
‘white'	t = 0 h	4	8	Cy5	2
‘white'	t = 0 h	1	1	Cy3	2
‘white'	t = 0 h	2	5	Cy5	2
‘white'	t = 0 h	3	6	Cy5	2
