# Supplementary material for: Blue light induces major changes in the gene expression profile of the cyanobacterium Synechocystis sp. PCC 6803
Source: Physiol Plant. 2020 Mar 14;170(1):10–26. doi: 10.1111/ppl.13086 (PMC7496141; doi:10.1111/ppl.13086)
Supplement: Supplementary file 5 — Table S3. Genes that were differentially expressed during the study and their annotation. [file PPL-170-10-s005.pdf]

Supplementary Table S3: Genes that were differentially expressed during the study and their annotation. Genes expressed with a log_2 fold change of <-1.4 or >1.4 and $p < 0.05$ were considered differentially expressed. We used Cyanobase (http://genome.kazusa.or.jp/cyanobase) as a guideline to distinguish between genes of different functional categories. We have also designated several genes that are listed as unknown or hypothetical in CyanoBase, but are annotated in the Kyoto Encyclopedia of Genes and Genomes (KEGG) (https://www.kegg.jp/) or in recent literature. In the latter case, references are added. References not included in the main text are listed as footnotes.

**ORF ID**	**Gene name**	**Time after light switch**										**Functional category and gene function (Cyanobase, KEGG, recent literature)**	**Reference (if not annotated in Cyanobase or KEGG)**
		blue light					orange light		red light		red dilute		
		1h	4h	24h	48h	192h	4h	192h	4h	192h	192h		
**Photosynthesis and respiration**													
sll1326	*atpA*	-0.76	-1.54	-1.64	-0.64	-0.42	-0.11	-0.25	-0.14	-0.19	-0.20	ATP synthase alpha chain	
sll1327	*atpC*	-0.74	-1.45	-1.39	-0.72	-0.27	-0.03	-0.15	-0.07	-0.10	-0.04	ATP synthase gamma chain	
sll1325	*atpD*	-0.78	-1.64	-1.68	-0.69	-0.48	-0.09	-0.34	-0.05	-0.13	-0.12	ATP synthase delta chain of CF(1)	
slr1330	*atpE*	-1.03	-1.53	-1.50	-0.64	-0.35	0.00	-0.39	0.32	0.23	0.54	ATP synthase epsilon chain of CF(1)	
sll1324	*atpF*	-0.92	-1.81	-1.80	-0.74	-0.53	-0.04	-0.38	0.01	-0.14	-0.15	ATP synthase B chain (subunit I) of CF(0)	
sll1323	*atpG*	-1.00	-1.74	-1.89	-0.76	-0.61	-0.09	-0.38	-0.01	-0.12	-0.13	ATP synthase subunit b' of CF(0)	
ssl2615	*atpH*	-0.75	-1.34	-1.70	-0.77	-0.58	-0.06	-0.28	-0.01	-0.15	-0.26	ATP synthase C chain of CF(0)	
sll1031	*ccmM*	-1.15	-1.48	-1.75	-1.16	-0.59	0.15	-0.07	-0.02	0.02	0.16	Carbon dioxide concentrating mechanism protein CcmM, putative carboxysome structural protein	
sll1032	*ccmN*	-0.94	-1.41	-1.37	-1.02	-0.35	0.19	-0.17	0.08	-0.10	0.40	Carbon dioxide concentrating mechanism protein CcmN, putative carboxysome assembly protein	
sll1471	*cpcG2*	3.61	3.38	3.63	2.85	2.69	3.81	2.80	3.33	2.47	2.74	Phycobilisome rod-core linker polypeptide CpcG2	
ssl0452	*nblA1*	2.52	2.34	2.47	1.31	1.23	0.27	0.80	0.04	0.41	0.45	Phycobilisome degradation protein NblA	
ssl0453	*nblA2*	1.98	1.84	1.82	0.59	0.68	0.32	0.88	0.02	0.17	0.15	Phycobilisome degradation protein NblA	
slr1687	*nblB2*	1.80	1.66	1.41	1.74	1.60	-0.28	-0.08	0.01	0.01	-0.20	Phycobilisome degradation protein NblB	Li and Sherman 2002
slr1311	*psbA2*	1.43	2.36	2.12	2.40	2.47	-0.18	0.15	0.13	0.17	-0.21	Photosystem II D1 protein	
sll1867	*psbA3*	1.39	2.30	2.10	2.40	2.45	-0.22	0.12	0.16	0.17	-0.25	Photosystem II D1 protein	

slr0906	*psbB*	0.91	1.61	1.13	1.03	0.83	-0.11	0.30	-0.03	0.06	-0.26	Photosystem II core light harvesting protein	
sll0849	*psbD*	1.01	1.82	0.94	1.22	0.96	-0.37	0.06	-0.12	-0.06	-0.40	Photosystem II reaction center D2 protein	
slr0927	*psbD2*	1.14	2.07	1.45	1.75	1.44	-0.27	0.10	-0.03	-0.03	-0.32	Photosystem II reaction center D2 protein	
**Other known categories and functions**													
sll1473	*CcaS; hik32*	1.31	1.25	1.69	1.19	1.08	1.85	1.19	1.71	0.89	1.29	Hypothetical; two-component sensor histidine kinase Hik32	Hirose et al. 2008
sll1475	*CcaS; hik32*	0.77	0.81	0.95	0.92	0.87	1.91	0.98	1.51	0.96	1.22	Regulatory functions; two-component sensor histidine kinase Hik32	
slr0473	*cph1, hik35*	1.14	2.16	2.15	1.10	0.83	-0.20	0.78	-0.48	0.06	0.22	Regulatory functions; cyanobacterial phytochrome 1, two-component sensor histidine kinase Hik35	
slr1251	*cyp*	-1.12	-1.69	-1.31	-1.57	-0.63	-0.10	-0.59	0.08	-0.03	0.21	Translation; protein modification and translation factors; peptidyl-prolyl cis-trans isomerase	
slr0676	*cysC*	-1.28	-1.82	-1.55	-1.31	-0.82	0.07	-0.42	0.40	0.01	-0.12	Amino acid biosynthesis; serine family/sulfur assimilation; adenylylsulfate kinase	
sll1441	*desB*	-1.24	-1.27	-0.85	-1.57	-0.61	-0.32	-0.95	0.46	-0.24	-0.25	Fatty acid, phospholipid and sterol metabolism; acyl-lipid desaturase (omega-3)	
sll0662	*Fed7*	0.68	1.23	1.49	1.62	1.03	-0.08	0.12	-0.10	-0.10	-0.50	Hypothetical protein; 4Fe-4S type iron-sulfur protein ferredoxin 7	Marteyn et al. 2009[1]
sll1009	*FrpC*	1.28	2.20	2.72	2.07	1.32	0.12	1.16	-0.28	0.66	0.00	Unknown protein; iron-regulated protein	
sll0145	*frr, rrf*	-1.16	-1.30	-1.41	-0.91	-0.75	-0.08	-0.22	-0.29	-0.17	-0.51	Translation; protein modification and translation factors; ribosome releasing factor	
slr1604	*ftsH*	1.66	1.55	1.24	1.16	1.12	-0.26	-0.27	-0.15	0.13	-0.25	Cellular processes; cell division protein FtsH	
slr0228	*ftsH*	1.53	1.55	1.08	1.40	1.20	-0.11	-0.21	0.08	0.15	-0.05	Cellular processes; cell division protein FtsH	
ssl1911	*gifA*	1.56	2.23	3.06	2.21	2.54	-0.46	-0.15	-1.27	-0.58	0.17	Hypothetical protein; glutamine synthetase inactivating factor IF7	García-Domínguez et al. 1999[2]
sll1515	*gifB*	1.23	1.95	2.52	2.44	2.42	-0.26	-0.59	-1.15	-0.75	-0.11	Unknown protein; glutamine synthetase inactivating factor IF17	García-Domínguez et al. 1999[2]
slr1124	*gpmB*	-1.35	-1.62	-1.31	-0.60	-0.60	-0.05	-0.39	0.41	0.27	0.03	Energy metabolism; glycolysis; phosphoglycerate mutase	
slr1992	*gpx2*	1.42	0.72	0.21	0.60	0.66	-0.19	-0.66	0.08	-0.09	0.05	Biosynthesis of cofactors, prosthetic groups, and carriers; glutathione peroxidase-like NADPH peroxidase	
slr2076	*groEL1*	-1.99	-3.26	-2.99	-1.72	-1.01	-0.09	-0.44	0.13	-0.23	-0.06	Cellular processes; 60kD chaperonin	
sll0416	*groEL-2*	-1.67	-2.75	-2.70	-1.38	-0.76	-0.02	-0.22	0.33	-0.03	0.02	Cellular processes; 60 kDa chaperonin 2, GroEL2, molecular chaperone	
slr2075	*groES*	-1.87	-3.25	-2.94	-1.63	-0.95	-0.09	-0.47	0.03	-0.21	0.01	Cellular processes; 10kD chaperonin	
ssr2595	*hliB, scpD*	3.31	2.57	2.12	1.55	2.26	-0.01	0.25	0.41	0.11	0.10	Other categories; adaptations and atypical conditions; high light-inducible polypeptide HliB	Promnares et al. 2006; Yao et al. 2007
ssl1633	*hliC, scpB*	1.52	1.18	0.93	0.52	0.72	0.04	-0.16	0.27	-0.09	-0.09	Hypothetical; high light-inducible polypeptide HliC	Promnares et al. 2006; Yao et al. 2007

sll0576	*HrEpiB*	-1.22	-1.83	-1.84	-1.28	-0.73	-0.11	-0.28	-0.12	-0.34	-0.42	Other categories; putative sugar-nucleotide epimerase/dehydratease	
sll1514	*hspA*	0.84	1.40	2.13	1.41	0.99	-0.86	0.25	0.15	-0.13	-0.45	Cellular processes; 16.6 kDa small heat shock protein, molecular chaperone	
sll0430	*htpG*	-1.06	-1.59	-0.91	-1.02	-0.47	0.03	-0.19	0.02	-0.11	-0.02	Cellular processes; heat shock protein 90, molecular chaperone	
slr1544	*lilA*	3.08	2.56	1.95	1.48	2.00	0.18	0.11	0.55	0.20	0.10	Unknown protein; member of the extended light-harvesting-like (Lil) protein family	Kufryk et al. 2008
slr1165	*met3*	-1.35	-1.86	-1.73	-1.30	-0.67	-0.17	-0.59	0.21	-0.06	0.14	Amino acid biosynthesis; serine family/sulfur assimilation; sulfate adenylyltransferase	
sll0657	*mraY*	-1.30	-1.40	-1.42	-0.87	-0.81	-0.06	-0.42	-0.08	0.00	-0.42	Cell envelope; murein sacculus and peptidoglycan; phospho-N-acetylmuramoyl-pentapeptide-transferase	
sll1450	*nrtA*	-0.30	-0.69	-1.55	-1.06	-1.32	0.28	-0.03	0.39	0.25	-0.23	Transport and binding proteins; nitrate/nitrite transport system substrate-binding protein	
slr0743	*nusA*	-0.75	-1.16	-1.45	-1.22	-0.54	-0.05	-0.57	0.16	-0.10	0.34	Transcription; RNA synthesis, modification, and DNA transcription; similar to N utilization substance protein	
slr1734	*opcA*	1.01	1.43	0.75	0.36	0.12	0.11	0.42	0.00	0.46	-0.03	Energy metabolism; pentose phosphate pathway; glucose 6-phosphate dehydrogenase assembly protein	
sll1694	*pilA1*	2.66	2.74	2.85	1.60	1.13	0.44	1.28	-0.26	0.73	0.49	Cellular processes; pilin polypeptide PilA1	
sll1695	*pilA2*	2.28	1.95	2.56	0.67	0.85	0.51	1.01	0.03	0.50	0.87	Cellular processes; chemotaxis; pilin polypeptide PilA2	
sll1696		1.46	0.84	1.55	0.39	0.60	0.50	0.62	0.13	0.29	1.29	Hypothetical; part of *pilA1,2* operon	Bhaya et al. 2000
slr2015	*pilA9*	1.11	1.23	1.67	1.31	1.13	0.15	0.04	0.18	0.36	0.44	Unknown protein; type 4 pilin-like protein, essential for motility	Chandra et al. 2015
slr2016	*pilA10*	1.46	1.64	2.38	2.00	1.55	0.34	0.31	0.17	0.52	0.59	Unknown protein; type 4 pilin-like protein, essential for motility	Chandra et al. 2015
slr2017	*pilA11*	1.38	1.75	2.63	2.08	1.60	0.43	0.53	0.28	0.50	0.84	Unknown protein; type 4 pilin-like protein, essential for motility	Chandra et al. 2015
slr2018		1.22	1.53	2.09	1.88	1.38	0.29	0.36	0.26	0.53	0.47	Unknown protein; part of *pilA* operon *sll2015-2019*	Chandra et al. 2015
slr1622	*ppa*	-1.11	-1.68	-1.40	-0.86	-0.63	-0.19	-0.45	-0.10	-0.07	-0.28	Central intermediary metabolism; Phosphorus compounds; soluble inorganic pyrophosphatase	
sll0680	*pstS*	-0.98	-1.69	-1.62	-1.12	-0.79	-0.39	-0.31	-0.63	0.00	-0.23	Transport and binding proteins; phosphate-binding periplasmic protein precursor (PBP)	
slr2132	*pta*	0.61	1.18	1.43	0.48	0.62	-0.16	0.15	-0.30	-0.25	0.26	Energy metabolism; pyruvate and acetyl-CoA metabolism; phosphotransacetylase	
sll0144	*pyrH*	-0.96	-1.07	-1.42	-0.97	-0.76	-0.08	-0.38	-0.11	-0.17	-0.52	Purines, pyrimidines, nucleosides, and nucleotides; uridine monophosphate kinase	
sll1744	*rpl1*	-0.60	-1.64	-1.68	-1.74	-0.65	0.08	-0.36	0.12	-0.17	0.21	Translation; ribosomal proteins: synthesis and modification; 50S ribosomal protein L1	
sll1745	*rpl10*	-0.04	-1.52	-1.73	-1.59	-0.67	0.04	-0.19	0.01	-0.21	0.12	Translation; ribosomal proteins: synthesis and modification; 50S ribosomal protein L10	
sll1746	*rpl12*	-0.45	-1.61	-1.93	-1.78	-0.75	0.16	-0.18	0.09	-0.09	0.04	Translation; ribosomal proteins: synthesis and modification; 50S ribosomal protein L12	

sll1806	*rpl14*	-1.21	-2.53	-2.39	-1.76	-0.77	-0.09	-0.41	0.05	-0.18	0.30	Translation; ribosomal proteins: synthesis and modification; 50S ribosomal protein L14
sll1813	*rpl15*	-0.86	-1.88	-1.87	-1.23	-0.72	-0.04	-0.28	0.22	-0.02	0.40	Translation; ribosomal proteins: synthesis and modification; 50S ribosomal protein L15
sll1805	*rpl16*	-1.31	-2.45	-2.37	-1.74	-0.71	-0.08	-0.44	0.19	-0.14	0.41	Translation; ribosomal proteins: synthesis and modification; 50S ribosomal protein L16
sll1811	*rpl18*	-0.89	-2.03	-1.89	-1.32	-0.68	-0.09	-0.30	0.08	-0.15	0.28	Translation; ribosomal proteins: synthesis and modification; 50S ribosomal protein L18
sll1802	*rpl2*	-1.60	-2.52	-2.58	-1.81	-0.89	-0.04	-0.38	0.17	-0.06	0.42	Translation; ribosomal proteins: synthesis and modification; 50S ribosomal protein L2
sll1803	*rpl22*	-1.34	-2.38	-2.40	-1.59	-0.72	-0.12	-0.41	0.12	-0.19	0.38	Translation; ribosomal proteins: synthesis and modification; 50S ribosomal protein L22
sll1801	*rpl23*	-1.45	-2.42	-2.37	-1.78	-0.79	-0.02	-0.39	0.14	-0.07	0.38	Translation; ribosomal proteins: synthesis and modification; 50S ribosomal protein L23
sll1807	*rpl24*	-1.16	-2.47	-2.30	-1.67	-0.65	-0.07	-0.43	0.09	-0.19	0.34	Translation; ribosomal proteins: synthesis and modification; 50S ribosomal protein L24
ssl3436	*rpl29*	-1.22	-2.48	-2.25	-1.66	-0.67	-0.08	-0.41	0.10	-0.17	0.35	Translation; ribosomal proteins: synthesis and modification; 50S ribosomal protein L29
sll1799	*rpl3*	-1.49	-2.28	-2.34	-1.72	-0.71	0.08	-0.31	0.27	-0.01	0.40	Translation; ribosomal proteins: synthesis and modification; 50S ribosomal protein L3
sll1800	*rpl4*	-1.42	-2.31	-2.42	-1.66	-0.81	-0.03	-0.41	0.21	-0.12	0.28	Translation; ribosomal proteins: synthesis and modification; 50S ribosomal protein L4
sll1808	*rpl5*	-1.08	-2.38	-2.20	-1.74	-0.65	-0.04	-0.39	0.12	-0.18	0.37	Translation; ribosomal proteins: synthesis and modification; 50S ribosomal protein L5
sll1810	*rpl6*	-0.87	-2.07	-1.91	-1.33	-0.61	-0.08	-0.27	0.19	-0.17	0.34	Translation; ribosomal proteins: synthesis and modification; 50S ribosomal protein L6
sll1818	*rpoA*	-0.85	-1.59	-1.51	-1.14	-0.54	0.00	-0.35	0.23	-0.03	0.38	Transcription; RNA synthesis, modification, and DNA transcription; RNA polymerase alpha subunit
sll1101	*rps10*	-0.85	-1.44	-0.89	-1.26	-0.34	0.16	-0.31	0.29	-0.04	0.20	Translation; ribosomal proteins: synthesis and modification; 30S ribosomal protein S10
ssl3437	*rps17*	-1.18	-2.46	-2.30	-1.69	-0.67	-0.05	-0.43	0.12	-0.16	0.37	Translation; ribosomal proteins: synthesis and modification; 30S ribosomal protein S17
ssl3432	*rps19*	-1.45	-2.46	-2.56	-1.67	-0.83	-0.09	-0.38	0.11	-0.14	0.39	Translation; ribosomal proteins: synthesis and modification; 30S ribosomal protein S19
sll1260	*rps2*	-0.59	-1.78	-1.42	-1.17	-0.60	0.04	-0.30	0.19	-0.08	0.15	Translation; ribosomal proteins: synthesis and modification; 30S ribosomal protein S2
sll1804	*rps3*	-1.41	-2.48	-2.52	-1.69	-0.82	-0.11	-0.44	0.13	-0.17	0.40	Translation; ribosomal proteins: synthesis and modification; 30S ribosomal protein S3
sll1812	*rps5*	-0.86	-1.89	-1.72	-1.44	-0.70	-0.04	-0.19	0.12	-0.10	0.25	Translation; ribosomal proteins: synthesis and modification; 30S ribosomal protein S5
sll1767	*rps6*	-0.70	-1.44	-1.15	-1.02	-0.41	0.00	-0.09	-0.05	0.01	-0.16	Translation; ribosomal proteins: synthesis and modification; 30S ribosomal protein S6

sll1809	*rps8*	-0.97	-2.28	-2.04	-1.59	-0.63	-0.10	-0.32	0.11	-0.16	0.41	Translation; ribosomal proteins: synthesis and modification; 30S ribosomal protein S8	
sll1688	*thrC*	0.65	1.41	1.59	0.63	0.26	0.28	-0.50	0.50	-0.13	-0.87	Amino acid biosynthesis; aspartate family; threonine synthase	
sll1261	*tsf*	-0.72	-1.37	-1.51	-1.33	-0.71	0.29	-0.34	0.40	0.07	0.04	Translation; protein modification and translation factors; elongation factor TS	
slr0798	*ziaA*	0.72	1.08	1.41	0.53	0.43	0.24	0.20	0.09	-0.02	-0.02	Transport and binding proteins; zinc-transporting P-type ATPase (zinc efflux pump) involved in zinc tolerance	
slr0915		1.33	1.73	1.97	1.91	1.35	0.20	0.43	-0.25	0.08	-0.34	DNA replication, restriction, modification, recombination, and repair; putative endonuclease	
slr1166		-1.19	-1.57	-1.34	-0.79	-0.31	-0.08	-0.45	0.09	-0.04	0.37	Energy metabolism; sugars; UDP-glucose:tetrahydrobiopterin glucosyltransferase	
slr1853		-1.04	-1.33	-1.55	-1.57	-1.41	0.04	0.04	0.01	-0.01	-0.31	Other categories; carboxymuconolactone decarboxylase	
sll1255		-0.04	0.68	1.52	-0.37	-0.45	0.42	0.61	0.59	0.16	0.12	Putative transposase [ISY203_c]	
slr1635		-0.02	0.59	1.41	-0.38	-0.47	0.36	0.57	0.53	0.11	0.14	Putative transposase [ISY203_e]	
slr0180		-0.04	0.65	1.48	-0.35	-0.48	0.35	0.60	0.56	0.08	0.10	Putative transposase [ISY203_f]	
sll1474		-0.06	0.60	1.45	-0.39	-0.47	0.37	0.60	0.55	0.09	0.16	Putative transposase [ISY203_g]	
slr1682		0.11	0.78	1.83	-0.03	-0.42	0.49	0.55	0.56	-0.12	0.07	Putative transposase [ISY391_b1]	
slr1859		-1.09	-1.49	-1.18	-1.48	-0.96	-0.05	0.12	-0.49	-0.19	-0.12	Transcription; RNA synthesis, modification, and DNA transcription; anti-sigma f factor antagonist	
sll0224		-0.83	-1.44	-1.71	-1.27	-0.89	-0.01	-0.02	-0.10	-0.13	-0.43	Transport and binding proteins; amino-acid ABC transporter binding protein	
**Hypothetical and unknown proteins**													
sll0263		-1.04	-1.55	-0.83	-1.31	-0.28	0.00	-0.84	0.27	-0.02	0.27	Unknown protein	
sll0473		0.82	1.09	1.37	1.42	0.60	0.02	0.29	-0.39	-0.04	0.12	Unknown protein; NitT/TauT family transport system substrate-binding protein	
sll0661		1.08	1.90	1.73	1.66	1.04	0.10	0.37	-0.01	0.21	-0.80	Hypothetical protein; YCF35	
sll0685		1.06	1.12	1.46	0.79	0.57	0.02	0.01	-0.03	0.13	0.07	Hypothetical protein; thioredoxin-like protein	
sll1109		-0.85	-1.45	-1.26	-1.15	-0.66	0.09	-0.24	-0.09	-0.12	-0.12	Hypothetical protein	
sll1158		1.34	1.75	2.29	2.11	1.38	0.07	0.60	-0.06	-0.03	-0.53	Hypothetical protein	
sll1193		1.17	1.64	1.64	0.94	0.84	0.04	0.43	-0.12	-0.19	-0.04	Hypothetical protein	
sll1338		1.20	1.67	1.16	0.52	0.78	0.12	0.85	-0.13	0.10	-0.28	Unknown protein	
sll1472		3.31	2.78	3.54	2.53	2.57	3.29	2.36	2.87	1.80	2.93	Unknown protein; part of *ccaS* gene cluster	Hirose et al. 2008
sll1526		-1.17	-1.67	-1.50	-0.80	-0.74	-0.22	-0.48	-0.52	-0.47	-0.73	Hypothetical protein; methyltransferase	
sll1722		-1.35	-1.50	-0.74	0.01	-0.16	-0.49	-0.55	0.72	-0.22	0.29	Hypothetical protein	
sll1862		1.00	1.05	2.99	2.27	2.47	0.18	0.50	-0.13	-0.19	0.17	Unknown protein	

sll1863	1.31	1.68	3.64	2.17	2.90	0.53	0.47	0.14	0.25	0.06	Unknown protein	
slr0082	1.56	0.98	2.24	1.27	1.83	0.50	0.34	0.04	-0.24	1.08	Hypothetical protein; ribosomal protein S12; methylthiotransferase	
slr0144	0.55	1.79	1.67	0.68	0.50	-0.31	0.50	-0.61	0.08	-0.19	Hypothetical protein; L-myo -inositol 1-phosphate synthase	
slr0179	1.07	1.57	1.82	1.05	0.88	-0.26	0.34	-0.31	0.12	-0.25	Hypothetical protein	
slr0364	1.93	2.07	2.41	1.43	1.25	-0.63	-0.68	-0.44	-0.54	0.99	Hypothetical protein	
slr0366	1.66	1.96	1.95	1.21	1.01	-0.77	-0.66	-0.42	-0.63	1.08	Unknown protein	
slr0373	4.40	5.37	4.97	4.28	2.52	0.24	1.25	0.04	0.66	-0.21	Hypothetical protein; part of operon *slr0373-slr0374-slr0376*	Singh and Sherman 2002
slr0374	4.11	5.01	4.56	3.85	2.34	0.30	1.17	0.22	0.62	0.03	Hypothetical protein; cell division cycle protein; part of operon *slr0373-slr0364-slr0376*	Singh and Sherman 2002
slr0376	3.36	4.57	5.04	3.81	3.09	0.63	1.66	0.50	0.95	-0.03	Hypothetical protein; part of operon *slr0373-slr0374-slr0376*	Singh and Sherman 2002
slr0404	0.85	1.50	1.20	1.26	0.84	-0.04	0.15	0.12	0.21	0.27	Hypothetical protein	
slr0442	2.19	1.99	2.64	0.88	1.00	0.35	1.10	-0.23	0.43	0.88	Unknown protein	
slr0443	-1.09	-1.46	-1.09	-1.04	-0.35	-0.03	-0.43	0.10	-0.07	-0.65	Hypothetical protein	
slr0587	0.82	2.54	2.02	1.79	1.19	-0.49	-0.30	-0.43	-0.20	-0.04	Unknown protein	
slr0607	-1.11	-1.58	-1.76	-1.47	-1.03	-0.12	-0.59	-0.09	0.07	-0.31	Hypothetical protein	
slr0888	0.26	2.00	2.37	1.75	1.04	-0.62	0.83	-1.08	-0.26	-0.06	Hypothetical protein	
slr1152	0.49	2.08	2.41	1.08	1.18	-0.54	0.32	-0.68	-0.28	-0.51	Hypothetical protein	
slr1436	-1.29	-1.93	-1.19	-1.43	-0.56	-0.28	-0.70	-0.54	-0.22	0.39	Unknown protein	
slr1583	0.96	1.89	1.73	0.77	0.93	-0.12	0.08	-0.43	0.00	-0.34	Hypothetical protein	
slr1634	2.02	2.97	3.23	3.29	2.20	-0.10	1.11	-0.74	0.13	-0.77	Hypothetical protein	
slr1674	1.40	1.81	1.90	0.53	1.11	0.18	0.38	0.54	0.39	-0.24	Hypothetical protein	
slr1770	1.21	2.03	1.88	1.76	0.99	0.23	0.72	-0.04	0.18	-0.35	Hypothetical protein	
slr1851	1.45	2.68	3.04	1.23	1.63	-0.18	0.15	-0.49	-0.05	0.03	Hypothetical protein	
slr1852	-0.28	-0.33	-0.80	-1.46	-1.15	0.14	0.15	0.00	0.06	-0.28	Unknown protein	
slr1917	1.41	1.96	1.52	0.96	0.78	-0.03	0.35	-0.18	0.25	0.36	Hypothetical protein	
slr1957	0.77	1.73	1.85	1.04	0.64	-0.39	0.55	-0.99	-0.43	-0.32	Hypothetical protein	
slr1958	0.41	0.88	1.58	0.89	0.76	-0.13	0.51	-0.32	-0.21	-0.07	Unknown protein	
ssl1263	1.23	2.05	1.91	1.74	1.12	0.09	0.37	-0.05	0.25	-0.77	Unknown protein	
ssl1533	1.05	1.44	0.84	0.25	0.35	0.02	0.44	-0.32	0.22	-0.08	Unknown protein	
ssl1762	1.63	2.26	1.92	1.15	0.74	0.18	0.45	0.03	0.13	-0.31	Hypothetical protein	
ssl2874	-0.68	-1.07	-1.45	-1.54	-0.94	0.11	-0.80	0.25	-0.23	-0.21	Hypothetical protein	

ssr0692	1.18	1.71	2.02	1.25	1.30	-0.32	-0.35	-0.61	-0.15	-0.35	Hypothetical protein
ssr1038	0.65	1.83	1.66	1.94	1.41	-0.16	-0.30	-0.75	-0.63	-0.86	Unknown protein
ssr1375	0.70	1.58	1.03	0.01	0.20	0.09	0.18	-0.06	0.08	-0.90	Hypothetical protein
ssr1562	1.09	2.03	1.76	1.11	0.89	-0.29	-0.08	-0.71	-0.29	-0.54	Hypothetical protein
ssr1853	-0.79	-1.25	-1.51	-0.98	-0.63	-0.17	-0.48	0.46	0.11	-0.18	Unknown protein
ssr2153	0.58	1.43	2.64	2.88	1.23	-0.30	0.66	0.67	0.18	-1.22	Unknown protein
ssr2848	1.12	1.50	1.46	1.22	0.60	0.25	-0.01	0.09	0.00	0.16	Unknown protein
ssr3129	1.20	2.08	2.38	0.63	0.89	-0.17	0.21	-0.32	-0.01	-0.04	Unknown protein

[1]Marteyn B, Domain F, Legrain P, Chauvat F, Cassier-Chauvat C (2009) The thioredoxin reductase-glutaredoxins-ferredoxin crossroad pathway for selenate tolerance in *Synechocystis* PCC6803. Mol Microbiol 71:520-532

[2]García-Domínguez M, Reyes JC, Florencio FJ (1999) Glutamine synthetase inactivation by protein-protein interaction. Proc Natl Acad Sci USA 96:7161-7166
